# Diet, exercise or diet with exercise: comparing the effectiveness of treatment options for weight-loss and changes in fitness for adults (18–65 years old) who are overfat, or obese; systematic review and meta-analysis

**DOI:** 10.1186/s40200-015-0154-1

**Published:** 2015-04-17

**Authors:** James E Clark

**Affiliations:** Division of Mathematics, Science, and Health Careers; Department of Science, Manchester Community College, Manchester, CT 06045-1046 USA

**Keywords:** Obesity, Exercise, Comparison, Weight loss

## Abstract

There are number of means of methods to alter body composition, and metabolic issues, available for the adult who is overfat. The following is a systematic review and meta-analysis focused on comparing changes from treatment program for adults who are overfat based on analysis of aggregated effect size (ES) of inducing changes. So as to determine the relative effectiveness of such protocols and intervention plans of choice. This tiered meta-analysis of 66-population based studies, and 162-studywise groups, a clear pattern of ES being established across and within treatments. First, hypocaloric balance is necessary for changing body composition, but the effectiveness for establishing imbalance does not equate with the effectiveness for body compositional changes, or any biomarkers associated with metabolic issues. With analysis showing that there is a necessity to include exercise in combination with diet effectively elicit changes in body composition and biomarkers of metabolic issues. More importantly, the combination, resistance training (RT) was more effective than endurance training (ET) or combination of RT and ET, particularly when progressive training volume of 2-to-3 sets for 6-to-10 reps at an intensity of ≥75% 1RM, utilizing whole body and free-weight exercises, at altering body compositional measures (ES of 0.47, 0.30, and 0.40 for loss of BM, FM, and retention of FFM respectively) and reducing total cholesterol (ES = 0.85), triglycerides (ES = 0.86) and low-density lipoproteins (ES = 0.60). Additionally RT was more effective at reducing fasting insulin levels (ES = 3.5) than ET or ET and RT. Even though generally lower ES than RT, the inclusion of ET was more effective when performed at high intensity (e.g. ≥70% VO_2max_ or HR_max_ for 30-minutes 3-4x’s/wk), or in an interval training style than when utilizing the relatively common prescribed method of low-to-moderate (e.g., 50-70% VO_2max_ or HR_max_ for at least equal time) steady state method, ES of 0.35, 0.39, and 0.13 for BM, FM, and FFM respectively. Thus indicating that focus of treatment should be on producing a large metabolic stress (as induced by RT or high levels of ET) rather than an energetic imbalance for adults who are overfat.

## Introduction

Accompanying the epidemic rise in the rate of obesity and obesity related diseases over the past half-century there has also been a rise in a variety of therapeutic interventions to address this epidemic. Most notable amongst these interventions have been numerous protocols that attempt to change body composition, most often through total mass reduction (i.e. weight loss). Resulting not only in a multibillion-dollar industry, but a greater absolute number of US adults currently engaging in behaviors (e.g., hypocaloric dieting, or involvement of general exercise and physical activity), with the focus based on the implication that all mass as being equal in the equation of body mass, obesity and disease [1-3]. To support such a position, several authors [4-9] have previously noted that there are limited differences in results for absolute changes in body composition with comparison between the various methodologies employed for treatment of weight issues for an individual who is overfat. While others [4,9-12] have indicated the responses are more related to an energetic imbalance (e.g., kcal/day, kJ/day) between dietary caloric load and expenditure from activity that results from the intervention of choice (e.g., diet, exercise, or combination therein) than the actual intervention for the adult who is overfat.

However, the discussion of effective outcome must go beyond any reduction in body mass or even health factors. Instead, the overall outcome has to involve a large change in self-selected and self-motivated behaviors. A change that serves to increase health and fitness behaviors and invoke a psychological adherence to exercise that most adults who are overfat might not intrinsically possess. Something that has become evident in the low attrition rates within many exercise programs and the high rate of repeated hypocaloric diet attempts [13-16]. Yet many of the marketing verbiage, seen through any scan of popular media, of intervention programs lead to the idea of adherence to any program appears related to the ability for that program to alter body mass. Which comes without mention or regard to any of the other physiological modifications, or alleviation of pathophysiological conditions, that arise throughout treatment that has been noted in the continuum of fitness and fatness factors impacting the overall health of the adult who is overfat [17-21]. This single focus on body mass alteration alone, leads to growing confusion within the general population as it relates to which therapeutic intervention may provide greatest benefit. Especially, given that there are any number of anecdotal, and single study results, indicating effectiveness of any of the various methods for weight loss and health improvement for the adult who is overfat. And more so are discussions of such reports within the scientific community, and popular press, which increase such confusion by indicating distinct advantages (or disadvantages) that are in conflict with each other. But also indicate the aforementioned limited differences between methods of intervention for the adult who is overfat.

Moreover, the differences between intervention methods used within single studies and the methods of comparison within previous reviews, lead to inherent issues of comparability of absolute changes between studies and the conclusion stipulated thereby [22,23]. In particular, the wide differences in the length of interventions and the vast elaboration and complexity within the design of some interventions utilized. Where most of the complexities that appear in some programs are at a level of elaboration for the sake of being elaborate, to function as a marketing ideal, and not based on the elaboration necessitated by principles of periodization and progressive training [24,25]. Likewise, the length of intervention time between comparisons varied greatly. Where, not surprisingly, the longer the intervention the greater absolute change relative to a shorter duration intervention. And taken together, may be the underlying rationale for the perplexing stats. Where even given an elevated current awareness of health issues of overfatness, there are reports by the Centers for Disease Control and Prevent (CDC) [1] identifying that fewer than 21% of US adults meet the general recommendation for exercise behaviors. And that only approximately 51% of US adults meet the recommendation for aerobic (endurance) training while only 29% meet the recommendation for strength (resistance) training each week [1,3].

Even if it is well understood that altering any health behaviors leads to a reduction in the risk factors for preventable non-communicable diseases [18,20,26-33]. And can lead to greater use of other healthy behaviors leading to greater overall levels of fitness [15,27]. Where these improvements appear to stem from a number of endocrinological changes that occur with both expression of overfatness and following exposure to exercise that ultimately alters the health status for the individual who is overfat [18,19,26,28,30,32-34]. The greatest impact of these changes appears to be related to alterations in sex hormone (i.e. testosterone and androgens), growth hormone, and a host of adipokines [29,34-46]. With low utilization of healthy behaviors eliciting changes indicated to increase the risk for the development of metabolic issues, which may culminate in Type 2 Diabetes Mellitus (T2DM), and are readily associated with reduced work capacity and anabolic hormone response for the individual who is overfat [18,26,27]. And are reversed with exposure to physical activity (e.g., exercise) with speculation that resistance exercise may provide the greatest impact on reversing such issues [28,47-50] and evidence for greater change in body composition from utilization of resistance exercise, both with and without conjunction with hypocaloric diet [51].

Given this level of understanding, it is perplexing that there would be such low investment in beneficial health behaviors that are highly associated with alleviating many of the aforementioned health issues [19,26,28,31]. Which leads to the question, if it is generally understood that physical activity is beneficial to not only in body mass reduction, in particular fat mass (FM) but not fat-free mass (FFM), along with improvements in many health functions, then why are so few adults engaging in such behaviors? As it has been reported that some 5-million U.S. deaths from non-communicable diseases could be prevented, even with a possible stagnation in the total proportion of the population classified as overweight or obese via current measures [1,2]. And may be related to the way in which exercise (in particular RT) is discussed in relation to the alteration of body mass, resolving metabolic issues and improvements in the overall health status for the adults who are overfat [20,21,52-54]. Along with the means by which we discuss changes elicited along with the process of comparison and generalization of findings to the population large. And a methodological bias in the employment of exercise that leads to an over recommendation of a single type of exercise based on personal preference [27,55-57]. Which, is compounded by the trove of anecdotal reports for response, from a self-professed exercise expert, that are easily accessible via any Internet search-engine for topics related to issue of weight loss.

However, just because there are issues related to direct comparison, due to methodological differences and taking into account the large inter-study discrepancies for responses, one can still compare responses. Comparison of responses must not come from the absolute value for changes indicated by each study, but as performed here through the aggregation of responses based on the pooled effect for ES that over the sum of all studies. Thus reflecting a more reliable overall effectiveness and provide a greater insight into the treatment phenomenon being offered [22,23]. Therein, the focus of this review here will examine the various outcomes from treatments utilized for improvements in the health status for the individual who is overfat that can be incorporated long-term behavioral modification. With analysis based on the effectiveness of treatment (e.g., effect size, ES) and not on the evaluation of absolute changes relative to either the initial state, or in comparison to a control group, within the included studies. Thus providing support to the health-care practitioner, or fitness club employee, to advice patients (or clients) as to which protocol schematics should provide the most effective means to not only change body composition (thus providing the reinforcement reward to elicit continual behavioral modification) but also improve the health status for the adult who is overfat.

Hence, the purpose of this systematic review is explore the current understanding of changes elicited to body composition in light of the understanding related to the endocrinological and health improvements seen with the various intervention programs based solely on population-based studies. That is related to treatment utilization of diet, diet and exercise or strictly exercise intervention for means of body mass reduction (i.e. weight loss), change in blood lipids and hormone levels. In an attempt to address the question if there is a difference in response between the various methods in (not absolute loss but effect size for) loss of body mass, fat mass and fat-free mass along with changes in blood lipid profiles and hormonal levels? Upon which, analysis will examine four distinct hypotheses. First, that exercise interventions will provide a greater effectiveness means for FM reduction than any diet intervention. Second, within exercise methods the use of RT will provide a stimulus that induces a greater effectiveness for change in body mass change (reduction in FM with retention of FFM) than ET, without regard to the addition of diet to the intervention. Thirdly, exercise will provide the stimulus that is more effective than any diet intervention at reverse hormone and adipokine/cytokine signals to normal “healthy” ranges. Lastly, that RT will be able to produce an effectiveness of treatment that matches the effectiveness of treatment from ET for both altering hormone and adipokine/cytokine signals but also for changing blood lipids.

## Methods

**Figure 1:**
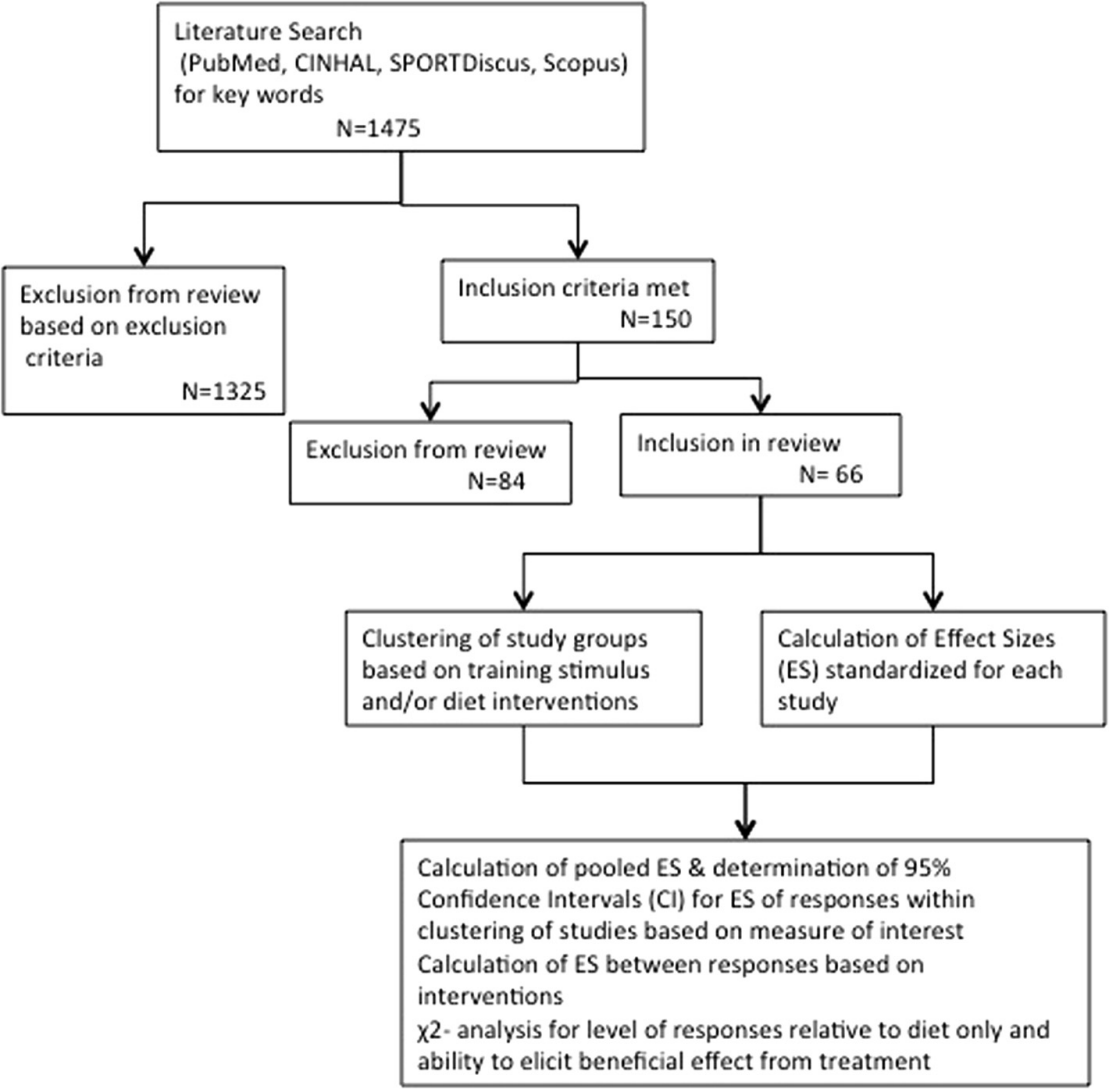
Description summarizing the steps for inclusion/exclusion and method leading to classification and analysis of studies involved within the meta-analysis.

As shown in the overview of the study in Figure 1, relevant studies (e.g., studies only involving human volunteers that fit into category of population based evidence) were retrieved from electronic database search engines (PubMed, EBSCO Host (CINAHL, SPORTDiscus) and Scopus) using the following key words in combination with each other: *obesity, exercise, resistance training, endurance training, strength training, aerobic training, diet, adipokine (adiponectin, leptin), cytokine (CRP,IL-1, IL-6, IL-10, TNF-*α*), anabolic hormone(testosterone, growth hormone),thyroid hormone, insulin, inflammation, weight loss, fat mass, and fat-free mass.* From the journal articles returned by the search engine, articles were included and excluded based on the following criteria. Additional studies were determined to be included for review based on citations within relevant articles.

Inclusion criteria:

- Published original research from January 1980-April 2013
- Published in English or translation of article available
- Utilized only human participants with reported average age for volunteers ranging from 18 and 65 years of age during the duration for the experiment
- Study population was either identified as either “overweight” or “obese” by authors or was indicated within the study as meeting at least 1 of the classification metrics for being overweight or obese (i.e. BMI > 25 kg/m^3^ or WHO levels of %BF for classification based on age and gender)
- Studies compared at least two conditions (either within subject cross-over design or comparison to a control or basal/baseline) and involved random assignment to training group(s) or control and to the order or method of training
- Study designs examined chronic adaptations (i.e. multiple training sessions, or interventions lasting at least 4 weeks in duration)
- Main purpose was to examine hormonal or cellular responses to exercise or diet
- Main purpose was to examine changes in body mass in response to exercise or diet
- Main purpose was to examine chronic responses to either exercise modes (e.g., resistance exercise or endurance exercise), hypocaloric diet, or combination of one of the exercise modes with hypocaloric diet, or combination of both exercise modes with hypocaloric diet.

Exclusion criteria:

- Publication was a review article
- Not published in English or no translation available
- Study design utilized an animal model for the problem
- Population age could be classified as adolescent, or juvenile, (average age < 18 years of age) and/or elderly (average age > 65 years of age)
- Study population either failed to meet metrics for classification as “obese” or “overweight”, or was indicated to have secondary disease (e.g., cancer, osteoporosis, cardiovascular disease) or had populations indicated to have history of metabolic variables and concurrent treatments (e.g., smoking, pharmacologically controlled type-2 diabetes mellitus (T2DM), cardiovascular diseases) that might confound the response to exercise and/or diet treatment
- Study design did not randomly assign subjects to a training group or control, or order of intervention
- Study design examined strictly acute responses (i.e., single exercise bout, or intervention lastly fewer than 4 weeks in duration)
- Main purpose did not involve measure of hormonal or cellular response to exercise or diet
- Results did not report absolute changes in hormones or body mass following intervention
- Indication of use of dietary supplement, or pharmacological dosing of anabolic or androgenic hormones.

Following retrieval and evaluation for inclusion, study data (reported means and standard deviation/error for measures of interest, number of subjects in each study groups, duration of study) were entered into database for subsequent analysis, see Figure 1. From the initial abstracts screened, a total of 66 studies were included in the meta-analysis, from which 162 study groups were included for comparison of responses within the review. Each included study was then classified by parameter of measurement and method, along with categorization of the method, of therapeutic intervention, see Table 1, for pooling and tabulating data for analysis based not only on the outcome of measure but for demographic information. From this pooled data for treatment responses averages, standard deviations were calculated across the studies classified by therapeutic intervention and measure of interest regardless of duration of intervention or any additional unique characteristics of the individual studies. Following which, pooled ES and confidence intervals (CI_.95_) of ES for each measure of interest was determined to examine the overall effect relative to a case of no change (i.e. CI_.95_ crossing zero within the 95% of all expected scores) based on each of the following comparisons, 1) relative to diet-only interventions; 2) relative to combination of diet and ET interventions; 3) relative to the combination of diet and RT interventions; and 4) relative to the combination of diet with ET and RT interventions.

**Table 1:** **Summary of studies include in meta-analysis indicating the therapeutic intervention used, and the principle measure of interest reported used for comparison within analysis**

**Study**	**Therapeutic intervention (Group (N) size & gender of treatment group)**	**Duration**	**Summary description of therapeutic intervention**	**Measures of Interest reported for comparison**
Ahmadizad [83]^$^	ET (8-M)	3x’s/wk for 12-wk	ET: 75–85% of MHR for 20-30-min (progressive),	BM, I, Adip
	RT (8-M)		RT: 4x12 CRT of 11 exercises @ 50–60% 1RM	
Anderssen [84]	D (34-M)	ET: 3x’s/wk for 52-wks	D: Low Fat	BM, FM, FFM, Cal
	ET (34-M)		ET: 60–80% of PHR for 60-min	
	D(E) (43-M)			
Ara [85]	RT (12-M)	RT: 3x’s/wk for 6-wks	RT: 1–3 x 3–12 @ Progressive 1RM (range 50-90%) for Squats, Leg Press, Leg Curl/Ext, Hip Flexion w/ 90 s rest @ total expenditure of 220–300 kcal/session	BM, FM, FFM, T, OB
Ballor [86]	ET (9-M)	3 x’s/wk for 12-wk	ET: 50% VO_2_max x 20–60 min (progressive)	BM, FM, FFM
			RT: 3x8 @ 50-80% 1RM (progressive) Squat, Bench, Leg Ext/Curl, Arm Ext/Curl, Lateral Pulldown	
	RT (9-M)			
Ballor [87]	D (10-W)	RT: 3x’s/wk for 8-wks	D: hypocaloric @ -1000 kcal/day w/ Protein >1.0 g/kg	BM, FM, FFM, Cal
	RT (10-W)			
	D(R) (10-W)			
			RT: 3x10-12 @ 10RM for: Chest Press, Leg Press, Lateral Pull-down, Arm Curl/Ext, Leg Curl/Ext, Calf Raise	
Borg [88]	D (90-M)	D: 2-month ET&RT: 3x’s-wk for 24-wk	D: Hypocaloric @ =1200 kcal/d for first and last wk and −500 kcal/d between	BM, FM, FFM, Cal
	ET (25-M)			
	RT (28-M)		ET: 45 min @ 60-70% VO_2_max	
			RT: 3x8 @ 60-80% 1RM CRT	
Bouchard [51]	D (11-W)	RT: 3x’s/wk for 12-wk	D: Hypocaloric @	BM, FM, FFM
	RT (11-W)		−500 kcal/d	
	D(R) (12-W)		RT: 3x8 @ 80% 1RM for (leg press, chest press, leg extension, shoulder press, sit-up, seated row, triceps extension, arm curl, and calf extension) w/ 60–90 s rest	
Brehm [89]	D, LF (20-W)	24-wks	D,LF: Hypocaloric @ ≈ 1250 kcal/d with ~54% CHO, ~18% protein, ~28% fat of kcal/d	BM, FM, FFM, TC, TG, LDL, HDL
			D, LC: Hypocaloric @ ≈ 1160–1300 kcal/d with ~15-30% CHO, ~25% protein, ~46-57% fat of kcal/d	
	D, LC (22-W)			
Brochu [90]	D (71-W)	RT: 3x’s/wk for 24-wk	D: Hypocaloric @ -500 kcal/d	BM, FM, FFM, TC, HDL, LDL, TG, CRP, I, Cal
			RT: 3–4 x 8–12 @ 65-80% 1RM (progressive) for (Leg Press, Chest Press, Lateral Pulldown, Shoulder Press, Arm Curl/Ext) w/ 60–90 s rest	
	D(R) (36-W)			
Bryner [91]	D(E) (2-M/8-W)	ET: 4x’s/wk RT: 3x’s/wk for 12-wk	D: Hypocaloric @ ≈ 800 kcal/d	BM, FM, FFM, Cal
			ET: 20–60 min (progressive) @ self-paced	
	D(R) (1-M/9-W)		RT: 2-4x15-12 @ 15RM-to-8-RM (progressive) for 10-exercise CRT w/ 60-s rest	
Campbell [92]^*$^	D (8-W)	RT: 3x’s/wk for 16-wk	D: Hypocaloric @ -500 kcal/d	BM, FM, FFM, Cal
			RT: 3x8-12 @ 80% 1RM (for Leg Ext/Curl, Leg Press, Chest Press, Arm Pull) w/ 60–120 s rest	
	D(R) (8-W)			
Christiansen [93]	D (29-M/W)	ET: 3x’s/wk for 12-wk	D: Hypocaloric @ ≈ 600 kcal/d	BM, I, OB, TC, HDL, Cal
	D(E) (25-M/W)			
			ET: 60–75 min @ unknown intensity to equate to 500–600 kcal/session	
	ET (25-M/W)			
Cuff [94]	D(E) (10-W)	3x’s/wk for 16-wk	E + R: 75-min @ 60-75% HRR w/ RT@ 2x12 for Leg Press, Leg Curl, Hip Ext, Chest Press, Latissimus Pulldown @ unknown intensity or rest E: 75 min @ 60-75% HRR	BM
	D(E + R) (9-W)			
Donnelly [95]^*^	D (26-W)	ET & RT: 4x’s/wk for 12-weeks	D ET: 20–60 min (progressive) @ 70% HRR RT: 2–3 x 6–8 @ 70-80% 1RM (progressive) on CRT exercises unknown, rest unknown	BM, FM, FFM, Cal
	D(E) (16-W)			
	D(R) (18-W)			
	D(E + R) (9-W)			
Donnelly [96]^*^	D (7-W)	RT: 3x’s/wk for 12-wks	D: Hypocaloric @ =700 kcal/d	BM, FM, FFM, Cal
			RT: 3 sets 8,6,6 @ 70% 1RM, progress to 4 sets 8.6.6.4 @ 80% 1RM for Bench Press, Latissimus Pull-down, Leg Ext/Curl, Shoulder Press, Arm Pullover, Arm Curl/Ext	
	D(R) (7-W)			
Donnelly [97]^$^	ET (16-M/25-W)	5x’s/wk for 68-wks	ET: 20–45 min @ 60%-75% HRR for 1^st^ 24-wks then 55%-70% of HRM (progressive) for ≈ 2000 kcal/wk (400 kcal/session)	
Dunstan [98]^*$^	D (17-M/W)	RT: 3x’s/wk for 24-wks	D: Hypocaloric	BM, FM, FFM, I, TC, HDL, LDL, TG, Cal
			RT: 3x8-10 @ 50-85% 1RM (progressive) for Bench Press, Leg Ext/Curl, Upright Row, Lateral Pull-down, Shoulder Press, Arm Curl/Ext, Abdominal exercises	
	D(R) (19-M/W)			
Fisher [99]^*$^	D (29-W)	ET & RT: 3x’s/wk for 8-wks	D: Hypocaloric @ ≈ 800 kcal/d	BM, FM, FFM, CRP, IL-6, TNF-α, Cal
			ET: 20–40 min @ 65-80% MHR (progressive)	
	D(E) (43-W)			
			RT: 1-2x10 @ 60-80% 1RM (progressive) for Leg Press, Squats, Leg Ext/Curl, Arm Curl, Lateral Pull-down, Bench Press, Military Press, Trunk Exercises	
	D(R) (54-W)			
Foster [100]^*^	D, HP (12-M/21-W)	52-wks	D, HP: Hypocaloric following *Dr. Atkins New Diet Revolution*	BM, FM, FFM, Cal
	D (8-M/22-W)		D: Hypocaloric @ M ≈ 1500–1800 kcal/d; W ≈ 1200–1500 kcal/d for 60%CHO, 15% protein, 25% fat	
Geliebter [101]	D (8-M/14-W)	ET & RT: 3x’s/wk for 8-wks	D: Hypocaloric @ <70% RMR	BM, FM, FFM, Cal
	D(E) (9-M/14-W)		ET: 8-min bicycle erg, 8-min arm erg, 8-min cycle erg @ 55-70% VO_2_peak (progressive)	
	D(R) (8-M/14-W)			
			RT: 2x6, 1xfatigue for Leg Ext/Curl, Chest Press, Arm Pull-over, Arm Curl/Ext, Leg Press w/30 s rest	
Goddpaster [102]	D (63-M/W)	ET: 5 d/wk for 24-wks	D: Hypocaloric @ ≈ 1200–2100 kcal/d with 50-55% CHO, 20-25% protein, 20-30% fat	BM, FM, FFM, Cal
	D(E) (67-M/W)			
			ET: total 60-min/d unknown intensity	
Hallsworth [103]	RT (11-M/W)	3x’s/wk for 8-wks	RT: 2–3 sets x unknown rep @ 50-70% 1RM (progressive) for: Arm Curl/Ext, Chest Press, Leg Curl/Ext, Lateral Pulldown, Shoulder Press	BM, I, TC, TG
Hammer [104]^*$^	D, LF (14-W)	ET: 5x’s/wk for 6-wks	D: hypocaloric VL @ =800 kcal/d, LF @ = 1195 kcal/d	BM, FM, FFM, Cal
	D, VL (12-W)			
	ET (12-W)		ET: distance of 1.6-4.8 km/session (progressive) @ 60-85% HRM (progressive)	
	D(E), LF (8-W)			
	D(E), VL (6-W)			
Hill [105]	D (3-W)	ET: daily for 5-wks	D: hypocaloric @ 800 kcal/d	BM, FM, FFM
			D(E): distance of 1.6-5.6 km/session (progressive) @ unknown intensity	
	D(E) (5-W)			
Hill [106]	D (6-W)	ET: 5x’s/wk for 12-wks	D: hypocaloric vary from 600–1500 kcal/d, LF @ 1200 kcal/d	BM, FM, FFM
	D, LF (8-W)			
	D(E) (10-W)			
	D(E), LF (8-W)		ET: 20–50 min (progressive) @ 60-70% HRM	
Ho [107]	D(E) (15-M/W)	ET & RT: 5x’s/wk for 12-wks	D: hypocaloric	BM, FM, FFM, I, OB, TC, HDL, LDL, TG, Cal
			ET: 30-min @ 60% HRR	
	D(R) (16-M/W)		RT: 4x12 @ 10RM for Leg Press, Leg Curl/Ext, Bench Press, Seated Row w/ 60 s rest	
	D(E + R) (15-M/W)			
			E + R: ET for 15-min @ 60% HRR & RT for 2x12 @75%1RM	
Ibanez [108]	D (12-W)	RT: 2-3x’s/wk for 16-wk	D: Hypocaloric @ -500 kcal/d	BM, FM, FFM, I, Adip, OB, TC, HDL, LDL, TG, Cal
			RT: 3-4x10-15 @ 50-80%	
	D(R) (13-W)		1RM (progressive) CRT for 8-wks & 3-5x10-12@60-80% or 3-5x 4-6@80-90% alternate for 8-wks	
Irving [109]^$^	E(Low-Intense) (3-M/10-W)	3-5 x’s/wk for 16-wks	Low-Intensity: unknown time @ RPE of 10–12 equate to 300–400 kcal/session	BM, FM, FFM, GH, IGF, HDL, TG, Cal
	E(High-Intense) (3-M/8-W)		High Intensity: unknown time @ RPE of 15–17 to equate to 300–400 kcal/session	
Josse [110]^$^	D(E + R), HP (30-W)	ET: 7x’s/wk RT: 2x’s/wk for 16-wks	ET: 7x’s/wk @ total expenditure of 250 kcal unknown duration or intensity	BM, FM, FFM, Cal, IL-6
	D(E + R), LP (30-W)			
	D(E + R), MP (30-W)			
			RT: 3x10 unknown intensity & rest interval	
Kempen [111]	D (10-W)	ET: 3x’s/wk for 8-wks	D: Hypocaloric @ =500-750 kcal/d	BM, FM, FFM, Cal
	D(E) (10-W)		ET: 90-min group exercise sessions @ 50-60% VO_2_max	
Kerksick [112]^$^	E + R (17-W)	ET&RT: 3x’s/wk for 14-wks	E + R: @ HR of 60-80% MHR using CRT of 14 exercises either paired:	BM, FM, FFM, Cal
	D(E + R), HC + P			
	(11-W)			
	D(E + R),VL/HP		Arm Ext/Curl, Leg Ext/Curl, Shoulder Press/Lateral Pulldown, Hip Abd/Add, Chest Press/Seated Row, Abdominal Crunch/Back Extension, Shoulder Shrug/Dip; or unpaired: Leg Press, Squat, Pec-Deck, Oblique, Hip Ext, side bends, stepping) x 30 s @ unknown %1RM w/ callisthenic 30 s between sets/paired exercise	
	(48-W) D(E + R),LC (37-W)			
	D(E + R),HC (41-W)			
Klimcakova [113]^$^	D(R) (12-M)	RT: 3x’s/wk for 12-wks	D: Hypocaloric	BM, FM, FFM, I, Adip, OB, TNF-α, TC, HDL, TG
			RT: 1x12-15 @ 60-70% for 17-exercise CRT	
Kraemer [114]	D (8-M)	RT & ET: 3x’s/wk for 12-wks	D: Hypocaloric	BM, FM, FFM, Cal
			ET: 30–50 min (progressive) @ 70-80% PHR	
			ET&RT: ET then, 1-3x5-10 @5-7RM or 8-10RM (alternate) for Squat, Military Press, Bench Press, Lateral Pull-down, Seated Row, Trunk exercises, Leg Press, Leg Curls, Calf Raises, Arm Curls with 60–180 sec rest (load dependent)	
	D(E) (11-M)			
	D(E + R) (10-M)			
Larsen [115]^*^	D, HGI/HP	Hypocaloric for 26-wks	All diets w/ < 25-30% total kcal from fat	BM, FM, FFM, Cal
	(97-M/W)			
	D, LGI/HP		LP: PRO:CHO ratio of 1:5 (10–15% total kcal protein and 57–62% total kcal CHO)	
	(124-M/W)			
	D, HGI/LP			
			HP: PRO:CHO ratio: of 1:2 (23–28% total kcal protein and 45–50% total kcal CHO)	
	(106-M/W)			
	D, LGI/HP			
			HGI: no change in GI diets w/ ~ 12% total kcal from protein	
	(124-M/W)			
			LGI: reduction of 15 GI points compared with the high-GI diets w/ ~ 12% total kcal from protein	
Layman [116]^*^	D, HP (12-W)	10-wks	D, HP: ≈1700 kcal/d @1.6 g/kg protein in ratio of CHO:protein ~1.4 and <30% fat of kcal/d	BM, FM, FFM, I, TH, TC, HDL, DLD, TG
	D, HC (12-W)		D, HC: ≈1700 kcal/d @0.8 g/kg protein and ratio of CHO:protein >3.5 and <30% fat of kcal/d	
Layman [117]^$*^	D, HP (12-W)	ET: 5x’s/wk RT: 2x’s/wk for 16-wks	D, HP: ≈1700 kcal @ 1.6 g/kg for protein with CHO:protein ratio <1.5 and fat <30% of kcal/d	BM, FM, FFM, Adip, OB, Ghrelin, I, TC, HDL, LDL, TG, Cal
	D(E + R), HP (12-W)		D, HC: ≈1700 kcal/d @ 0.8 g/kg for protein with CHO:protein ratio >3.5 and fat <30% of kcal/d	
	D, HC (12-W)			
	D(E + R), HC (12-W)			
			ET: 30-min @ unknown intensity	
			RT: 1x12 @ unknown resistance intensity for 7 exercise in CRT	
Maiorana [118]^$^	E + R (14-M/2-W)	3x’s/wk for 8-wks	E + R: CRT for RT @ 45 s of RT @ 55–65% MVC (progressive) w/ 15 s rest between RT followed by 5-min ET @ 70–85% PHR (progressive) intermittent to RT-exercises	BM, HDL, LDL, TC, TG
Marks [119]	D (10-W)	ET & RT: 3x’s/wk for 20-wks	D: Hypocaloric general low fat @ -628 kcal/d	BM, FM, FFM, Cal
			ET: 12–36 min (progressive) @ 70-85% HRM	
	D(E) (8-W)			
	D(R) (11-W)		RT: 2x8-12 @ 70-90% 1RM for: Leg Ext/Curl, Seated Row, Chest Press, Arm Ext/Curl, and abdominal curls, with unknown rest	
	D(E + R) (9-W)			
			ET&RT: 12–24 min of ET and 1 set of RT	
Moreira [120]^$^	E(S) (8-M/W)	3x’s/wk for 12-wks	E(S):20–60 min (progressive) @ 10% of Anaerobic Threshold	BM, FM, FFM, TC, TG
			E(I): 20–60 min (progressive) total time @ 2:1 ratio of 120% Anaerobic Threshold to Rest time	
	E(I) (8-M/W)			
Nicklas [121]	D (53-M/W)	ET & RT: 3x’s/wk for 72-wks	D: Hypocaloric @ -500 kcal/d	BM, CRP, IL-6, TNF-α, Cal
			ET: 30–45 min @ 50-75% HRR	
	ET (53-M/W)			
			E + R: 15-min ET @ 50-75% HRR, followed by 15-min RT @ 2x12 CRT, followed by 15-min ET @ 50-75% HRR	
	D(E + R) (53-M/W)			
Oberbach [122]^$^	ET (40-M/W)	4x’s/wk for 4-wks	ET: 3-days: 60-min unknown intensity (20-min calisthenics/20-min steady state/20-min “power-training” & 1-day: 60-min swimming	BM, FM, FFM, Adip, OB, IL-6, IL-10, CRP, TC, HDL, LDL, TG
Olson [123]	RT (16-W)	2x’s/wk for 52-wk	RT: 3x8–10 @ 8-10RM (Progressive) for unknown exercises indicated as isotonic variable resistance machines and free weights targeting the following major muscle groups: quadriceps, hamstrings, gluteals, pectorals, latissimus dorsi, rhomboids, deltoids, biceps and triceps	BM, FM, FFM, I, Adip, Il-6, CRP, TC, HDL, LDL, TG
Pavlou [124]^*$^	D (41-M)	ET: 3x’s/wk for 8-wks	D: Hypocaloric @ = 800 kcal/d	BM, FM, FFM
	D(E) (31-M)		ET: 70-85% MHR for 20–45 min (progressive)	
Phinney [125]	D, HP (6-W)	ET: 6-hrs/wk for 4-wks	D: hypocaloric @ =720 kcal/d	BM, FM, FFM, Cal
	D(E) (6-W)		ET: 6-hrs/wk total time @ 50% VO_2_max	
Polak [126]	ET (25-W)	5x’s/wk for 12-wks	ET: 45-min @ 50-65% VO_2_max (progressive) for 2x’s/wk group exercise class, 3x’s/wk cycle ergometer	BM, FM, FFM I, Adip, OB, IL-6, TC, HDL, TG
Pritchard [127]	D, LF: (18-M)	ET: 5x’s/wk for 52-wks	D: hypocaloric @ -500 kcal/d & 20-25% fat of kcal/d	BM, FM, FFM, Cal
	ET (21-M)		ET: 30–45 min @ 65-75% HRM	
Racette [128]	D (17-W)	ET: 3x’s/wk for 12-wks	D: hypocaloric @ =75% BMR	BM, FM, FFM, Cal
	D(E) (13-W)			
			D(E): 35-min @ 65% VO_2_max	
Rice [129]	D (9-M/W)	ET: 5x’s/wk RT: 3x’s/wk for 16-wks	D: Hypocaloric @ -1000 kcal/d	BM, FM, FFM
			ET: 20–60 min @ 50-85% MHR (progressive)	
	D(E) (10-M/W)			
			RT: 1x8-12 @ 8-12RM (progressive) for Leg Ext/Curl, Latissimus pull-over, Bench Press, Should Press, Arm Ext/Curl	
	D(R) (10-M/W)			
Rolland [130]^*^	D, HP (1-M/16-W)	36-wks	D,HP: hypocaloric @ 800–1500 kcal/d, @ 20%CHO,40% protein, 40% fat of kcal/d	BM, FM, FFM, I, Adip, OB, HDL, TC
	D, LF (5-M/9-W)		D, LF: hypocaloric @ -500 kcal/d @ 35%, CHO, 36% protein, 28% fat of kcal/d	
Ross [131]	D (15-W)	ET: daily x 14-wks	D: Hypocaloric @ -500 kcal/d	BM, FM, FFM, I, Cal
			ET: self-selected duration @ ~80% MHR (equate to 500 kcal/session)	
	D(E) (17-W)			
	ET (12-W)			
Ryan [132]^*$^	D (23-W)	ET & RT: 3x’s/wk for 24-wks	D: Hypocaloric @ -250-350 kcal/d	BM, FM, FFM
			ET: 45-min @ 50-75% HRR (progressive)	
	D(E) (24-W)			
	D(R) (16-W)		RT: variable resistance for 15-rep (3RM to 15 RM) 2–3 sets for Leg Press, Chest Press, Chest Flies, Latissimus Pull-down, Leg Curl/Ext, Arm Curl/Ext w/ 30 s rest	
Schjerve [133]^$^	E(S) (13-M/W)	3x’s/wk for 12-wks	IT: 10-min @ 50-60% MHR followed by 4 cycles of 4-min:3-min ratio of 85-95% MHR then 50-60% MHR followed by 5-min @ 50-60% MHR	BM, FM, FFM, TC, HDL, TG
	E(I) (14-M/W)			
	RT (13-M/W)		ET: 47-min @60-70% MHR	
			RT: 4x5 @ 90% 1RM (progressive) for Leg Press or Squats, trunk exercises @ 3x30 w/ 30 s rest	
Shue [134]	D (21-W)	12-wks	D: Hypocaloric @ -500-1000 kcal/d	I, Adip, OB, IL-6, IL-10, TNF-α, TC, HDL, LDL, TG
Sigal [135]	D(E) (60-M/W)	ET & RT: 3x’s/wk for 24-wks	ET: 15–45 min @ 60-75% MHR (progressive)	BM, FM, FFM, HDL, LDL, TG
	D(R) (64-M/W)		RT:2-3x7-9 @ unknown intensity in CRT w/ unknown exercises	
	D(E + R) (64-M/W)			
			E + R: combined both	
Slentz [136]	ET (22-M/26-W)	3x’s/wk for 32-wks	ET: equivalent to 12 mi/wk @ 75% VO_2_peak	BM, FM (as indicated by abdominal)
	RT (22-M/30-W)		RT:3x8-12 @ unknown RM in CRT fashion	
	E + R (19-M/25-W)			
			E + R: full sessions of both ET & RT	
Strasser [7]^*$^	D (10-W)	ET: 3 x’s/wk for 8-wks	D: Hypocaloric @ -500 kcal/d	BM, FM, FFM, TC, HDL, LDL, TG, Cal
	D(E) (10-W)		ET: 60-min @ 60% VO_2_max	
Tjønna [137]	E(I) (4-M/7-W)	3x’s/wk for 16-wks	IT: 10-min @ 70% MHR followed by 4-cyles of 4-min:3-min @ 90% MHR and 70% MHR, then 5-min @ 50-60% MHR	BM, FM, FFM, I, Adip, HDL, TG
	E(S) (4-M/4-W)			
			ET:47-min @ 70% MHR	
Tokmakidis [138]^$^	D(E + R) (9-W)	4x’s/wk (2x’s ET, 2 x’s RT) for 16-wks	ET: 2x’s/wk: 45-min @60-80% MHR (progressive)	BM, FM, FFM,I
			RT: 2x’s/wk: 3x12 @ 60% 1RM (progressive) for Bench Press, Row, Leg Ext/Curl, Latissimus, Pec Deck w/ 45–60 s rest Pull-down,	
Trapp [139]	E(I) (15-W)	3x’s/wk for 15-wks	IT: cycle ergometer @ 8-sec sprint:12-sec recover intervals progress from 5-min to 20-min total time	BM, FM, FFM,I, Adip, OB
	E(S) (15-W)			
			ET: 10–40 min @60% VO_2_peak (progressive)	
Volpe [140]^*$^	D (13-M/15-W)	ET: 3–5 x’s/wk for 36-wks	D: hypocaloric @ ≈ −500 kcal/d	BM, FM, FFM, OB, TC, HDL, LDL, TG, Cal
	ET (17-M/17-W)			
	D(E) (14-M/14-W)		ET: 15–30 min for 3–5 x’s/wk (progressive) @ unknown intensity via ski-ergometer	
Wang [141]^*^	D, HGI/HP(24-W)	8-wks hypocaloric and 24-wks 1 of 4 maintaining diets	D: 8-wks of low fat/ Hypocaloric @ =800 kcal/d & 24-wks of:	BM, I, TC, HDL, LDL, TG, Cal
			LF: < 25-30% total kcal from fat with compensatory increase in protein and CHO	
	D, LGI/HP (24-W)			
	D, HGI/LP (24-W)		LP: PRO:CHO ratio of 1:5 (10–15% total kcal protein and 57–62% total kcal CHO)	
			HP: PRO:CHO ratio: of 1:2 (23–28% total kcal protein and 45–50% total kcal CHO)	
	D, LGI/HP (24-W)			
			HGI: no change in GI diets w/ ~ 12% total kcal from protein	
			LGI: reduction of 15 GI points compared with the high-GI diets w/ ~ 12% total kcal from protein	
Watkins [142]	ET (14-M/W)	ET: 3–4 x’s/wk for 26-wks	D: hypocaloric @ ≈ 1200–1500 kcal/d w/ fat @ 15-20% total kcal/d	BM, FM, FFM, I, TC, HDL, LDL, TG, Cal
	D(E) (14-M/W)		ET: 30–35 min @ 70-80% HRR	
Wycherely [143]	D (16-M/W)	RT: 3x’s/wk for 16-wks	D: Hypocaloric @ ≈ 1200–1250 kcal/day w/ 0.7 g/kg protein	BM, FM, FFM, I, CRP, TC, HDL, LDL, TG
	D,HP (12-M/W)			
	D(R) (17-M/W)			
			D,HP: Hypocaloric @ ≈ 1200-	
			1250 kcal/day @1.2 g/kg protein	
	D(R), HP (14-M/W)		RT: 2x8-12@70-85% 1RM for Leg Press, Leg Ext, Chest Press, Latissimus Pull-down, Seated Row, Arm Ext w 60 s rest	

Note *denotes only treatment ES determined for diet-only intervention, ^$^denotes only treatment ES determined within exercise interventions. Legend: D = diet, RT = resistance training, ET = Endurance Training, E + R = combination of exercise, HP = high protein diet/low carb, HC + P = high carbohydrate & protein, HC = high carbohydrate/low fat, GI = glycemic indexed diet, HGI = high glycemic diet, LGI = low glycemic diet, LC = low carb/no protein change, LF = low fat (American Heart Assoc.), VL = very low caloric diet, LP = low protein, MP = moderate protein, D(R) = diet and resistance training, D(E) = diet and endurance training, D(E + R) = diet and combination of exercise, E(S) = steady state endurance, E(I) = interval endurance training, MHR = maximal heart rate; PHR = peak heart rate, HRR = heart rate reserve, CRT = circuit resistance training, IT = interval training, MVC = maximal volitional contraction, RPE = rating perceived exertion, TC = total cholesterol, HDL = high-density lipoproteins, LDL = low-density lipoproteins, TG = triglycerides, T = testosterone, GH = growth hormone, I = insulin, TH = thyroid hormones, IGF = insulin-like growth factor, Adip = adiponectin, OB = leptin, CRP = c-reactive protein, Cal = Caloric Expenditure or reduction from diet, M = men, W = women.

In order to complete comparisons between dissimilar experimental designs, all studies were evaluated for a standardized effect size (ES). Based on the premise for comparing ES previously utilized [58-60], for each of the measures of interest based on the therapeutic intervention (Figure 1). This standardized ES across all studies was undertaken in an effort to control for difference in methods of measurement and distinct (unique) qualified differences in the therapeutic interventions (see Table 1). And thus allow for comparison between and within the various parameters measured based on the therapeutic intervention in a pooled fashion of ES for response. Each of measure of interest and within all groups (interventions as well as indicated control) the treatment ES were calculated via (*μ*_post − treatment_ − *μ*_pre − treatment_)/(*σ*_pooled within_). After which, each measure of interest had a pooled ES determined between the various treatment protocol groupings and the control grouping to elicit the pooled therapeutic effect, via equation (*μ*_change treatment_ − *μ*_change control_)/(*σ*_pooled with control_). Additional comparison of the pooled ES for changes were made on between the responses noted in the various measures of interest across, and relative to the pooled response for the control groups, indicated in the studies included in the analysis, based on the equation, (*μ*_change across − treatment # 1_ − *μ*_change across − treatment # 2_)/(*σ*_pooled between treatment_). Following which, a standardized confidence interval (CI) for ES was calculated within each treatment intervention for use in the comparison of responses between interventions for each measure of interest based on the pooling of studies for comparison.

In an effort to establish a secondary directionality for difference between treatments, the within study treatment ES were then clustered for 2x2 χ^2^ analysis to determine if any difference in the level of response, standardized ES, by outcome based on the measure of interest for comparison between responses based on relationship (i.e. above or below) to the pooled ES for that given treatment, and compared between the type of treatment and then based on sub-classification of physical activity within the treatment (e.g., resistance exercise, RT, endurance exercise, ET, organized exercise program, or general physical activity program), type of diet (e.g., general hypocaloric, low-fat, or low-carbohydrate diet), the combination of the diet with exercise programs, and based on the length of intervention within the grouping of treatment.

## Results

### Pooled effects

As seen in Table 2, there are a wide variety of results that were obtained from each of the therapeutic interventions utilized. Not only for the reduction of body mass (including FM and FFM) but also for changes in adipokines, hormones, and blood lipid profiles. Such findings indicate that all treatments provide an effective means to elicit change relative to status at start of treatment or to the control treatment. Interestingly, there were differences noted between effects favoring the combination of diet with ET versus diet alone for alterations in body mass (χ^2^ = 3.09, p = 0.055). And differences indicate an effectiveness favoring for the combination of diet with RT versus diet along for reduction in FM (χ^2^ = 3.8, p < 0.05) and retention of FFM (χ^2^ = 6.7, p < 0.0001). With no significant difference noted between the effectiveness for diet with ET or diet with RT for alterations to BM, but a difference for effectiveness that favors RT over ET for FFM retention (χ^2^ = 10.15, p < 0.01). When examining the effect of ET, when separated from the aspect of additional diet intervention, ET alone appears to less effective to allow for the retention of FFM than dieting alone, or in a combination with use of diet (χ^2^ = 7.458, p < 0.01). While the use of RT, both alone as well as in combination with diet provides greater stimulus for retention of FFM (χ^2^ = 3.5, p < 0.05). There were also distinct differences noted in responses based on gender and the type of treatment utilized. Where males tend to have a larger pooled ES for responses to diet with RT retention of FFM and reduction in FM relative to female groups (χ^2^ = 3.94, and 3.64, p < 0.05, respectively). Along with males indicated has having a greater level of effectiveness relative to females for loss of FM and retention of FFM following an intervention of diet with combination of both ET and RT (χ^2^ = 3.64, p < 0.05). While females trended toward having a larger pooled ES for responses to diet alone, χ^2^ = 2.09 (p = 0.11), and in combination to ET for reduction in total body mass, χ^2^ = 1.94 (p = 0.12), and FM, χ^2^ = 3.1 (p = 0.09), but not FFM. Lastly, as related to changes in caloric (energetic) balance there were no differences noted between any of the pooled ES for the assumed differences in energetic balance across the various treatment interventions.

**Table 2:** **Summary of response based on the pooled therapeutic effect size (ES), from the 32 studies that indicated control group, ES (CI for ES), based on method of therapeutic intervention and measure of interest**

	**Pooled ES (CI)**					
**Body Masses:**	**D**	**D(E)**	**ET**	**D(R)**	**RT**	**D(E + R)**
**Body mass**	1.24 (0.25, 2.23)	1.19^$^ (0.14, 2.25)	0.2^¢^ (−0.38, 0.78)	1.06^#^ (0.07, 3.12)	0.25 (0.007, 0.42)	0.57 (0.29, 0.84)
**Fat mass**	0.88 (0.22, 1.53)	1.07^$^ (0.41, 1.73)	−0.16^¢^ (−0.80, 0.49)	0.63^#¢^ (0.13, 1.57)	0.36 (−0.30, 0.59)	0.14 (−0.86, 1.13)
**Fat-free mass**	0.48 (0.001, 0.95)	0.02^$^ (−1.05, 1.08)	0.80 (0.61, 0.99)	1.08*^#¢^ (0.61, 1.56)	2.23* (−1.5, 5.95)	0.20 (−0.18, 0.57)
**Hormones, Adipokines, Cytokines:**						
**Insulin**	0.30 (−0.03, 0.63)	0.55 (−0.24, 1.34)	0.11 (−0.49, 0.72)	0.47^#^ (0.01, 0.95)	0.79 (−0.81, 2.39)	0.30 (−0.03, 0.63)
**Adiponectin**	0.13 (−0.18. 0.43)	−0.84^$^ (−3.01, 1.33)	1.27 (−0.02, 2.54)	1.35^#^ (−0.66, 3.36)	1.05 (−0.05, 2.14)	
**Leptin**	−0.38 (−1.88, 1.11)		1.57 (1.25, 1.90)	1.07^#^ (0.46,1.67)		−0.38 (−1.88, 1.11)
**Blood Lipids:**						
**Total cholesterol**	0.39 (−0.13, 0.90)	0.16^$^ (−0.35, 0.67)	−0.16 (−0.67. 0.34)	0.32 (−1.13, 1.76)	0.001 (−0.22, 0.22)	0.93
**HDL**	0.11 (−0.14, 0.37)	0.38^$^ (−0.13, 0.90)	0.96 (0.31, 1.60)	−0.31*^#^ (−1.11, 0.48)		−0.19* (−1.00, 0.62)
**LDL**	−0.01 (−0.20, 0.18)	−0.09^$^ (−0.69, 0.50)	−0.30 (−0.33, −0.28)	0.04^#¢^ (−0.89, 0.96)		−0.45 (−1.46, 0.56)
**Triglycerides**	−0.05 (−0.20, 0.11)	0.14^$^ (−0.07, 0.24)	−0.28 (−0.81, 0.24)	0.24 (0.03, 0.45)	−0.27 (−0.48, −0.06)	0.61 (0.08, 1.14)
**Caloric difference**	0.48 (−0.15, 1.11)	0.49^$^ (−0.15, 1.13)	1.16*^$^ (0.15, 2.18)	0.19 (−0.50, 0.79)		0.48 (−0.15, 1.11)

Note that a negative ES favors the control intervention while a positive ES favors the therapeutic intervention and that for measures of changes to fat-free mass, the indication for retention of mass is considered to be positive. Cells left empty did not have enough responses to indicate either a pooled therapeutic ES relative to control or a CI for ES. Note that D indicates intervention of diet only, ET indicates endurance training, RT indicates resistance training, D(E) indicates intervention of diet with ET, D(R) indicates intervention of diet with resistance training, D(E + R) indicates intervention of diet with combination of training methods.

*Indicates significantly greater response than diet-only intervention, ^$^Indicates difference between modes of endurance exercise intervention, ^#^indicates difference between intensity used for resistance exercise intervention, ^¢^indicates difference in gender response (male > female), ^¢*^indicates difference in gender response (female > male), for χ^2^-value > χ^2^CV, p < 0.05.

In comparison of blood lipid profiles, e.g., total cholesterol (TC), high-density lipoproteins (HDL), low-density lipoproteins (LDL), and triglycerides (TG), all treatment options once again provided an effective means for change relative to either the pre-intervention status or in comparison to the control conditon. The responses invoked by RT, whether alone or in combination with diet, showed a greater effectiveness for eliciting changes in TC and LDL relative to the diet only options (χ^2^ = 7.18, 4.95, respectively) and trends toward significance for HDL (χ^2^ = 3.38, p = 0.068). But showed no difference to the responses invoked by ET, either with or without the combination of diet. While the use of ET either alone, or in combination with diet, show no difference for effectiveness at eliciting changes blood lipids (TC, LDL and TG) versus the changes elicited by a diet only intervention. Yet trended toward favoring ET for effectiveness in changes seen in HDL (χ^2^ = 2.842, p = 0.089). Additionally, there were no differences noted on the pooled effect for treatment based on the gender of the participant groups for any of the treatment intervention options. However there was a trend for women utilizing ET in combination with diet for having a great effect in the changes in HDL levels versus those seen in men (χ^2^ = 2.0, p = 0.12).

Similar to the changes seen with blood lipids, effectiveness for eliciting positive changes to adipokines (adiponectin and leptin) or cytokines (c-reactive protein (CRP), TNF-α) of interest were noted occurring from all treatment interventions. Furthermore, there were very few differences in the pooled effect size versus the diet only intervention, with difference in ES for changes of adiponectin and leptin being elicited by the use of RT, either with or without diet, trending toward significant difference, χ^2^ = 3.085 (p = 0.07) and χ^2^ = 3.45 (p = 0.06), respectively. While there were no differences noted between the therapeutic effectiveness for treatment in the responses to either CRP or TNF-α between any of the combinations for interventions.

### Comparison between treatment effects

**Figure 2:**
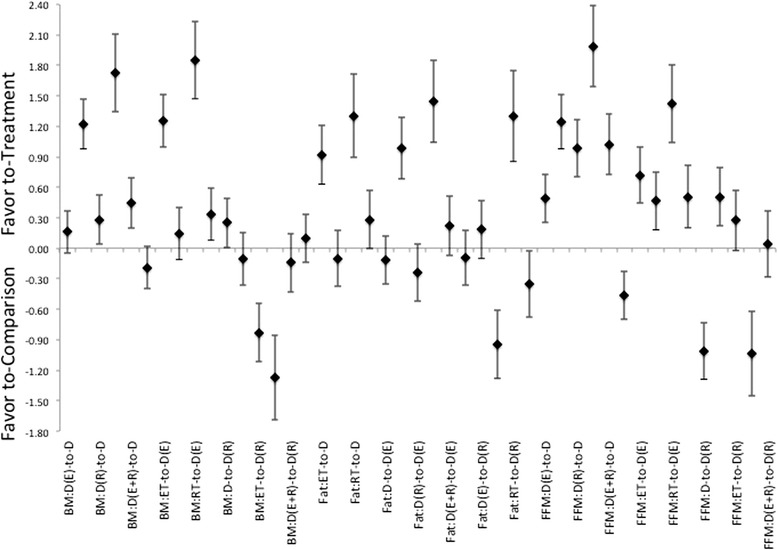
Description of the pooled ES for treatment response and the range of CI for ES between intervention (versus diet alone or versus diet with combination of ET, or versus diet with combination of RT) methods for changes in either Body Mass (BM), Fat Mass (Fat), and Fat-Free Mass (FFM). Note that the comparisons are labeled as “treatment-to-comparison”, with D indicating diet-only, D(E) indicating diet with ET, D(R) indicating diet with RT, D(E + R) indicating diet with ET and RT, ET indicating ET-only, and RT indicating RT-only for the various intervention methods within the comparisons.

In comparison of body compositional changes based on the method of intervention, as would be expected, there are effect size differences in treatment responses that favor the combination of intervention methods. While a diet alone treatment did induce a beneficial treatment effect following intervention. It was not more effective than other treatments at inducing changes in FM, see Figure 2. While the combination of diet and ET was not as effective as any of the other treatments with respect to changing of body composition. ET appears to be effective at inducing a larger loss of FFM relative to diet with combination of RT (χ^2^ = 6.531, p = 0.01). With respect to the combination of diet and RT, this intervention appears to be able to induce favorable adaptions in, measurements of both FM and FFM (χ^2^ = 9.24 and χ^2^ = 8.02, p < 0.01, respectively). While producing equivalent ES for body mass changes as either diet alone, or diet in combination with ET, see Figure 2. Interestingly, there were no differences noted showing a favor toward the combination of diet with both ET and RT versus the other intervention methods. In continuation with what was noted in the pooled therapeutic effect size, a trend toward gender difference for effectiveness of treatment was noted in the change in FFM for the utilization of diet with RT only in male groups versus female counterparts (χ^2^ = 3.3, p = 0.06).

**Figure 3:**
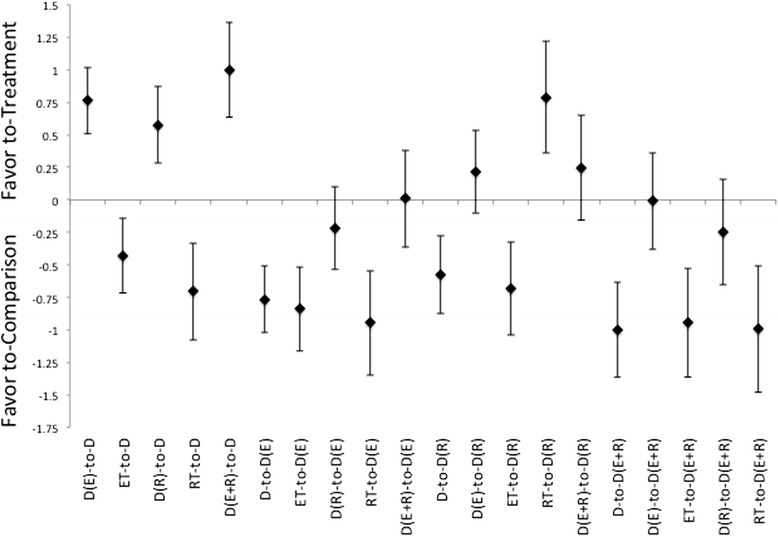
Description of the pooled ES for treatment response and the range of CI for ES between intervention (versus diet alone or versus diet with combination of either ET, RT, or combination of ET and RT) methods for changes in energetic imbalance as assumed established within the intervention protocol. Note that the comparisons are labeled as “treatment-to-comparison”, with D indicating diet-only, D(E) indicating diet with ET, D(R) indicating diet with RT, D(E + R) indicating diet with ET and RT, ET indicating ET-only, and RT indicating RT-only for the various intervention methods within the comparisons.

Eliciting energetic imbalance indicates a pattern that favors an intervention that is a combination of diet with any type of exercise versus that of either diet, or exercise, alone, Figure 3. Furthermore, the treatment ES for energetic imbalance for the combination of diet and ET were more favorable than any other treatment intervention combinations. Interestingly, while the combination of diet with ET and RT was more effective then either a diet alone or exercise alone it was less effective then either ET or RT in combination with diet at inducing an energetic imbalance. Additionally, there were no differences between gender groups that would indicate a greater effectiveness of a treatment methodology for a specific gender grouping.

**Figure 4:**
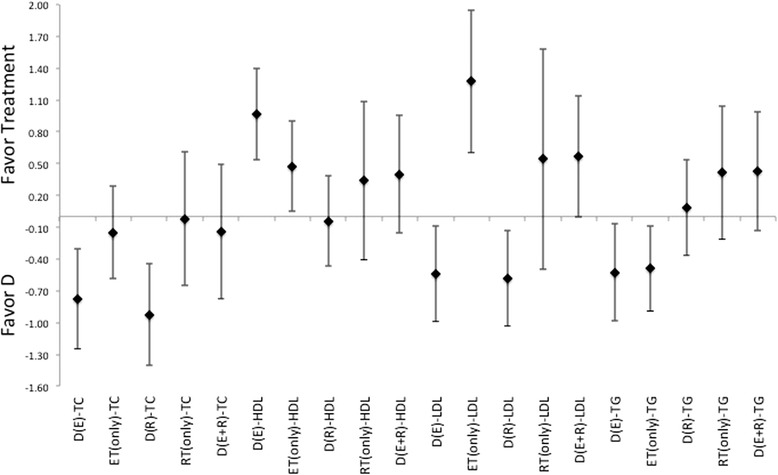
Description of the pooled ES for treatment response and the range of CI for ES between intervention (versus diet alone) methods for response related to changes in blood lipid profiles TC, HDL, LDL, and TG. Note that labeled groups go as follows: D indicating diet-only, D(E) indicating diet with ET, D(R) indicating diet with RT, and D(E + R) indicating diet with ET and RT for the various intervention methods within the comparisons.

**Figure 5:**
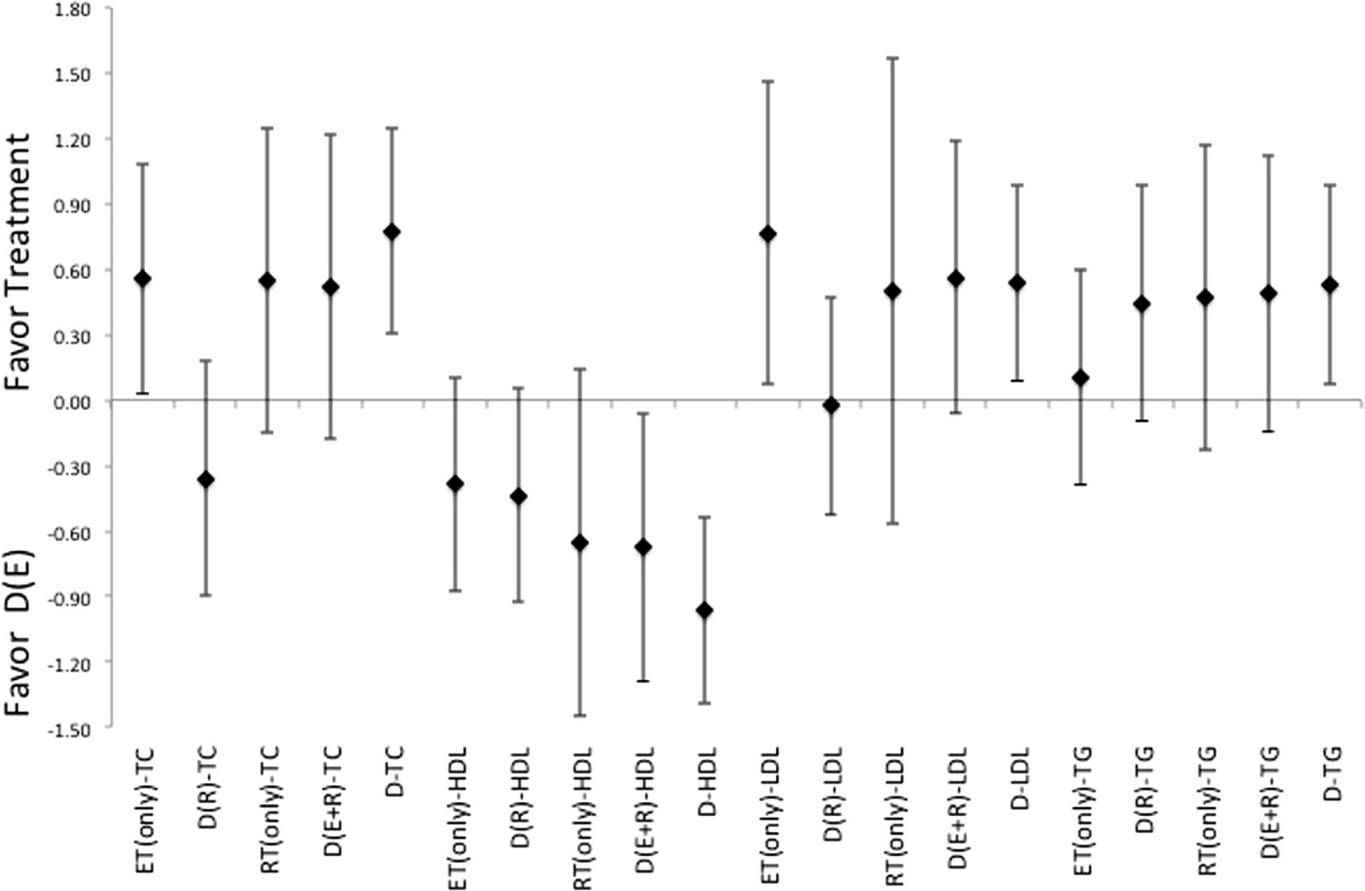
Description of the pooled ES for treatment response and the range of CI for ES between intervention (versus diet with combination ET) methods for response related to changes in blood lipid profiles TC, HDL, LDL, and TG. Note that labeled groups go as follows: D indicating diet-only, D(E) indicating diet with ET, D(R) indicating diet with RT, and D(E + R) indicating diet with ET and RT for the various intervention methods within the comparisons.

**Figure 6:**
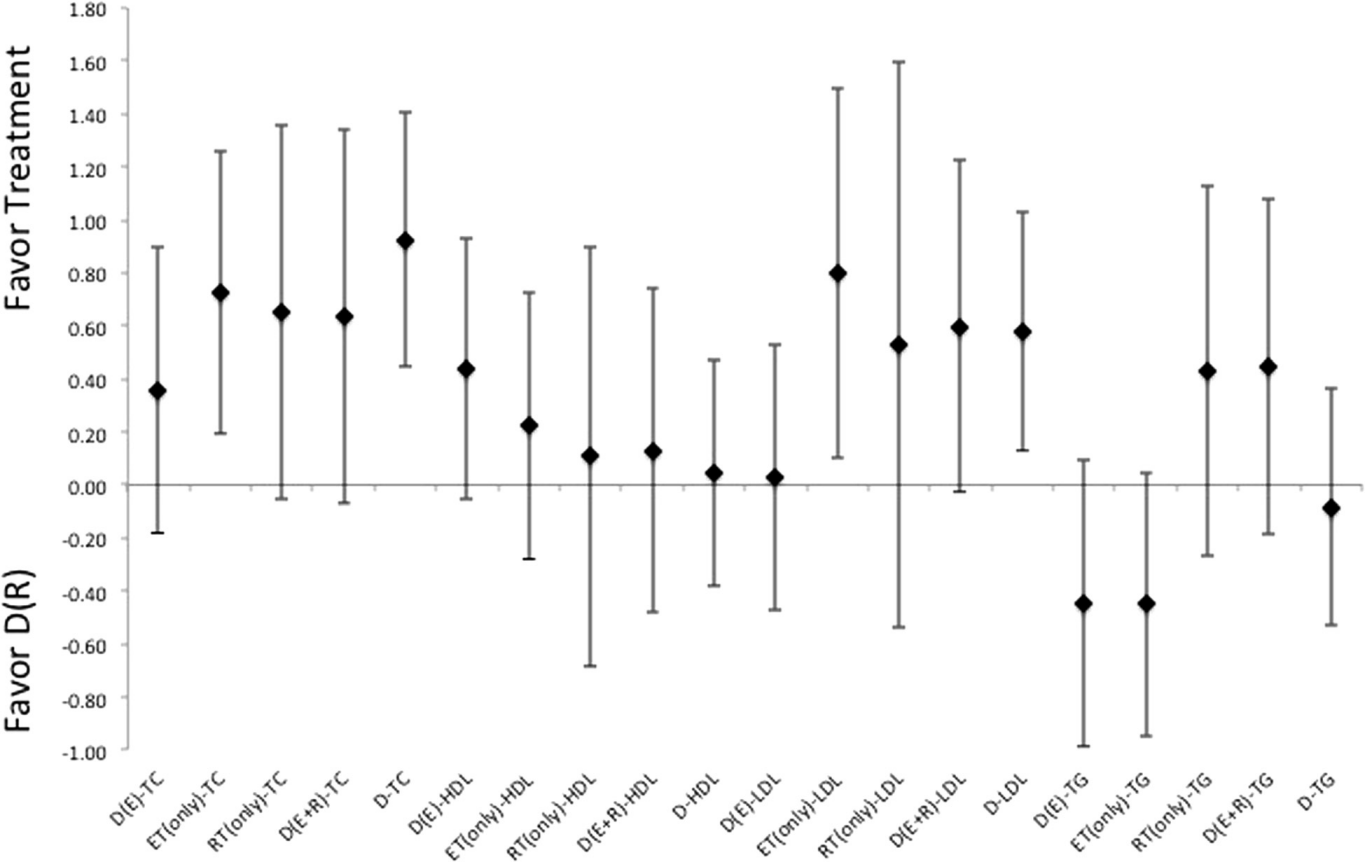
Description of the pooled ES for treatment response and the range of CI for ES between intervention (versus diet with combination of RT) methods for response related to changes in blood lipid profiles TC, HDL, LDL, and TG. Note that labeled groups go as follows: D indicating diet-only, D(E) indicating diet with ET, D(R) indicating diet with RT, and D(E + R) indicating diet with ET and RT for the various intervention methods within the comparisons.

In regards to changes in the blood lipid profiles, there were not only indications for difference between treatments, there is also a very interesting finding that therapeutic interventions may actually induce elevations in certain lipids. While the diet only intervention did have a positive impact on TC and HDL levels, it has only minimal impact on either LDL or TG levels, Figures 4, 5 and 6. Additionally, the treatment interventions that combined diet with ET induced a much larger ES, Figure 4, for measures TC and LDL. And diet in combination with RT induces a larger ES in TC, HDL, LDL and TG changes relative to those changes seen in diet only treatments, Figures 4, 5 and 6. Moreover, diet with combination of RT was able to produce a much lager ES for these measures in comparison to those induced by diet with combination of ET, for each of these measures, see Figures 4, 5 and 6. As far as changes in TG, diet with combination of RT appears be the least effective for inducing changes relative to either the diet only or the diet with combination of ET, Figures 4, 5 and 6. Additionally, there appears to be a pattern where the induction for changes in lipid profiles cannot be established through the use of ET only for all measures. While RT is the only intervention that appears to be slightly more effective than diet alone or diet in combination of ET for changes in HDL and TG, Figures 4, 5 and 6. There were gender differences noted for effectiveness of treatment for HDL and TG but not for LDL or TC, both of which indicate a larger effectiveness for treatment in female grouping versus male counterparts.

**Figure 7:**
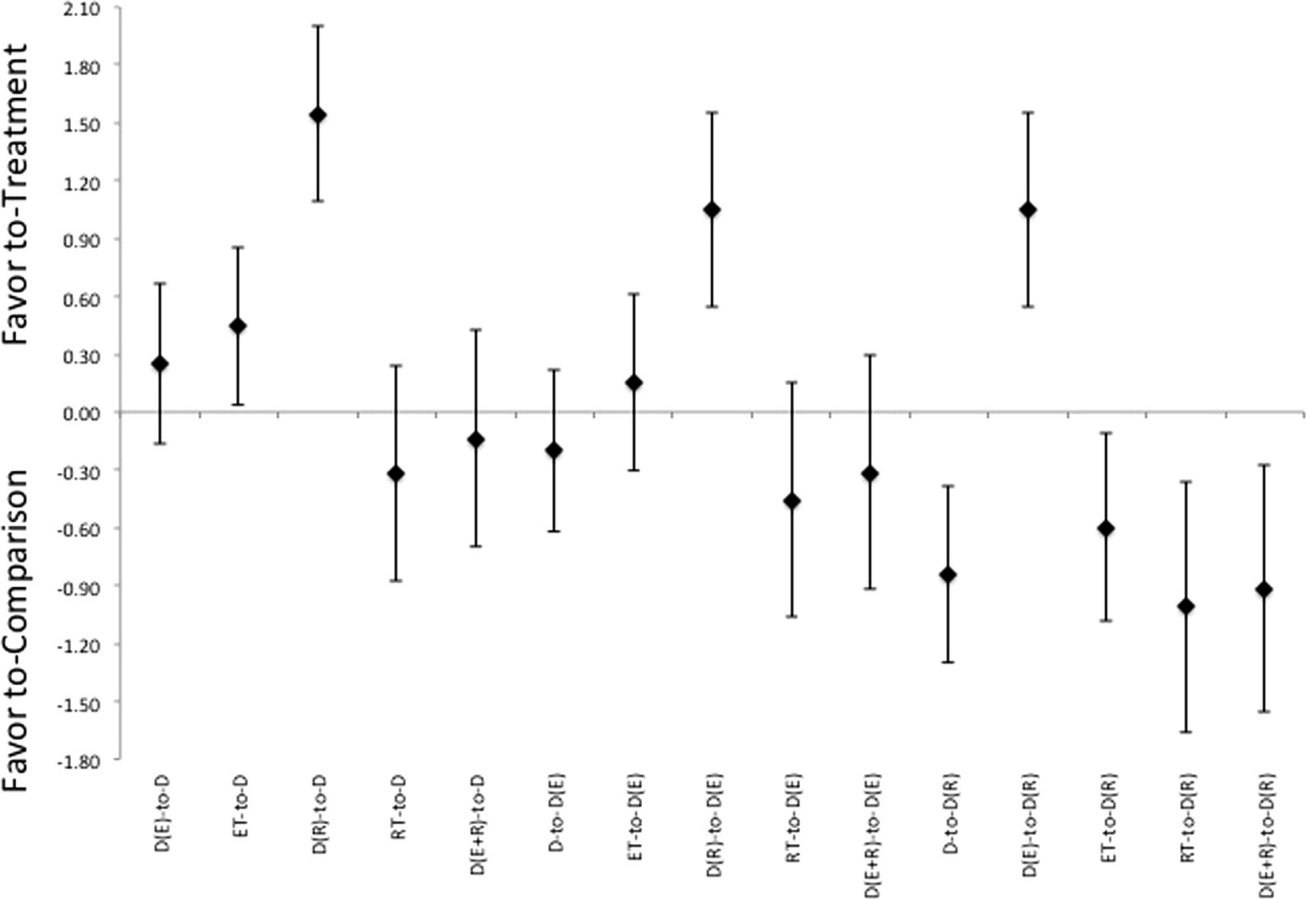
Description of the pooled ES for treatment response and the range of CI for ES between intervention (versus diet alone or versus diet with combination of ET, or versus diet with combination of RT) methods for response related to changes in Insulin. Note that the comparisons are labeled as “treatment-to-comparison”, with D indicating diet-only, D(E) indicating diet with ET, D(R) indicating diet with RT, D(E + R) indicating diet with ET and RT, ET indicating ET-only, and RT indicating RT-only for the various intervention methods within the comparisons.

The most prominently reported hormones and cytokine signal throughout the studies was insulin, followed by adiponectin, leptin, IL-6, CRP, and TNF-α. And as such are the hormone and cytokines reported on here as they provide a large enough N-size to allow for comparison of a pooled ES and CI for ES based on treatment intervention. In which ES for eliciting changes in insulin, Figure 7, indicates that ET in combination with diet (or as a stand-alone intervention) induces a lower effect than diet alone. While the use of RT either alone, or in combination with diet, was more effective than diet alone it was less effective than ET or the combination of diet with ET and RT, see Figure 7. There was a gender difference to response and effectiveness indicated within the analysis for insulin changes, with treatments appearing to be more effective in male groupings than in female groups.

**Figure 8:**
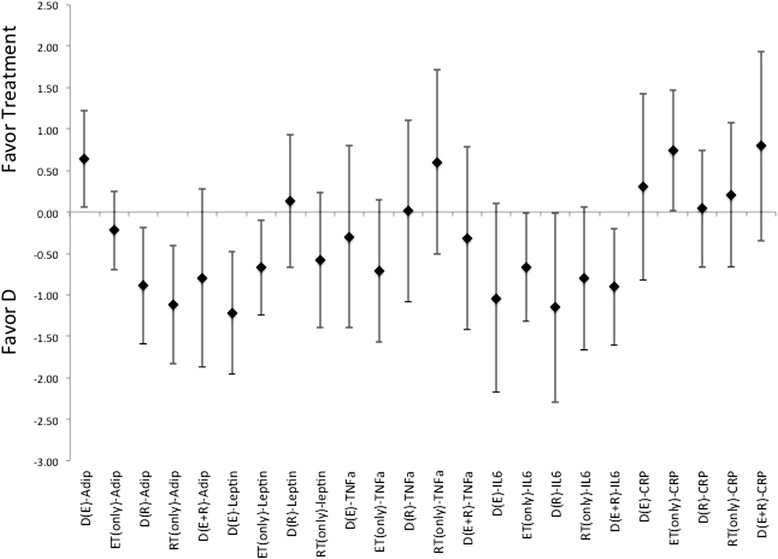
Description of the pooled ES for treatment response and the range of CI for ES between intervention (versus diet alone) methods for response related to changes in Adiponectin, Leptin, CRP, TNF- α and IL-6. Note that labeled groups go as follows: D indicating diet-only, D(E) indicating diet with ET, D(R) indicating diet with RT, and D(E + R) indicating diet with ET and RT for the various intervention methods within the comparisons.

**Figure 9:**
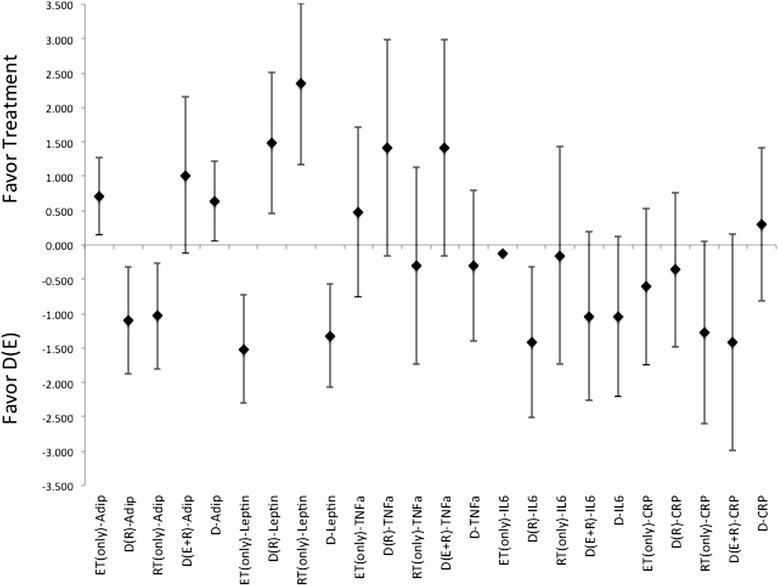
Description of the pooled ES for treatment response and the range of CI for ES between intervention (versus diet with combination of ET) methods for response related to changes in Adiponectin, Leptin, CRP, TNF- α and IL-6. Note that labeled groups go as follows: D indicating diet-only, D(E) indicating diet with ET, D(R) indicating diet with RT, and D(E + R) indicating diet with ET and RT for the various intervention methods within the comparisons.

**Figure 10:**
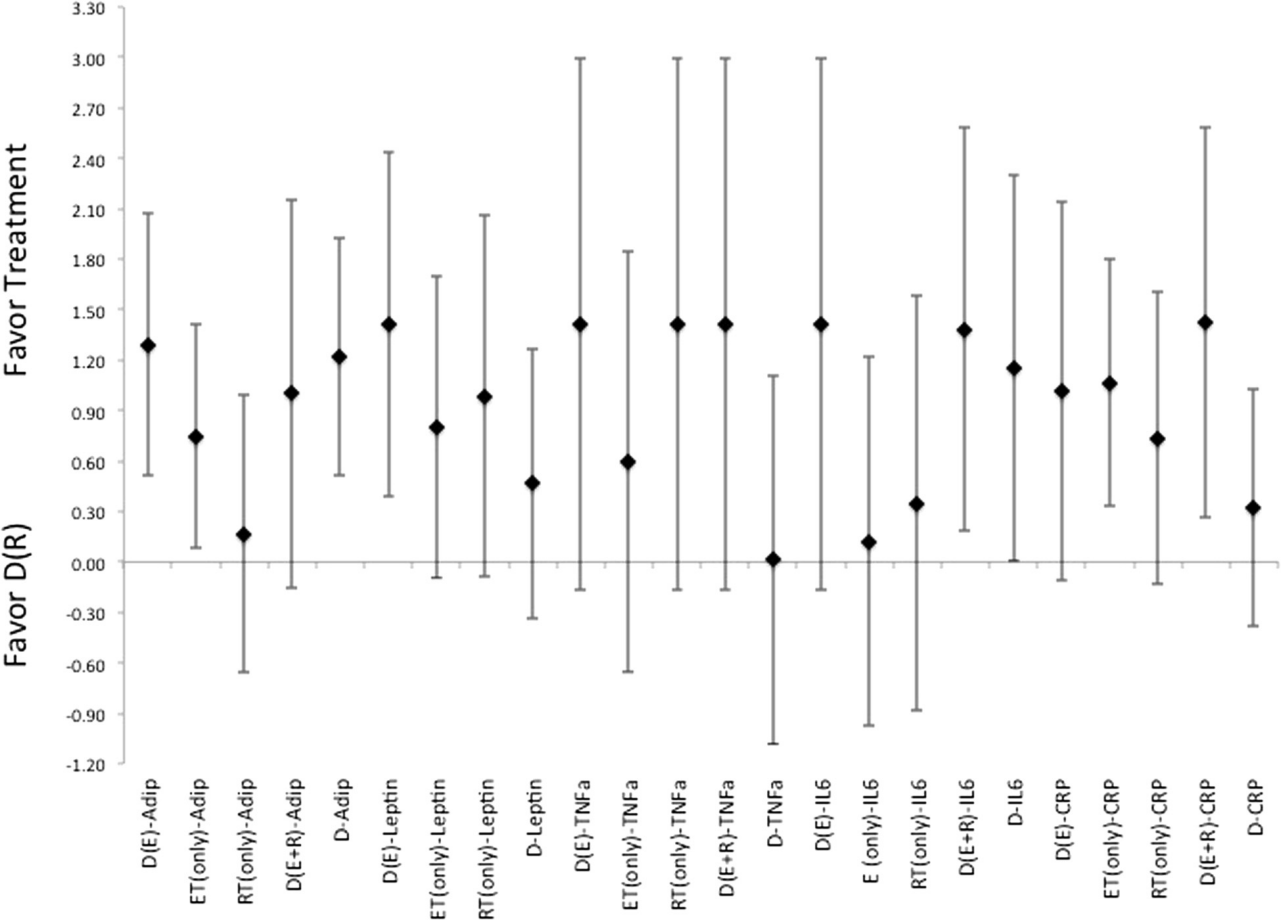
Description of the pooled ES for treatment response and the range of CI for ES between intervention (versus diet with combination of RT) methods for response related to changes in Adiponectin, Leptin, CRP, TNF- α and IL-6. Note that labeled groups go as follows: D indicating diet-only, D(E) indicating diet with ET, D(R) indicating diet with RT, and D(E + R) indicating diet with ET and RT for the various intervention methods within the comparisons.

There were highly variable responses for effectiveness for each treatment method to induce changes to circulating levels of adiponectin, leptin, TNF-α, or CRP (Figures 8, 9 and 10). In which, diet alone and in combination with ET, were more effective than what was seen with changes induced by the incorporation of RT for changes to adiponectin and leptin, Figures 8, 9 and 10. While the changes induced in CRP and TNF-α, Figures 8, 9 and 10, were nearly identical, i.e. ES that crosses 0, for differences in effectiveness for changes between diet alone, or diet in combination with exercise (either ET, RT or combination of ET and RT). And all were more effective than the exercise alone treatments. Further there were no indication for a more effective means to change cytokine or adipokine levels with the utilization of diet in combination with both ET and RT. Interestingly, there were no gender differences indicated throughout the analysis of ES for any of the changes to the level of cytokines or adipokines following treatments, regardless of the methodology employed.

### Comparison within treatment methods

Not only were there differences indicated between the treatment options, but also within the various treatment methods. First is the differences based on diet method. With the use of a high protein diet, indicated here as a diet with >1.5 g protein*kg^−1^body mass (>25% of total kcal/d), in a hypocaloric model inducing a larger effect for body compositional changes relative to any of the other diet methods, ES of 0.60, 0.54, 0.38 for loss of body mass, FM and retention of FFM respectively. Further, lower fat diet was less effective when compared to either a glycemically controlled diet, or the high protein/low carbohydrate diet for the change of any body compositional measures, ES of −0.64. Especially in relation to the high protein/low carbohydrate diet, ES of −1.04. Similarly, the lower carbohydrate and higher protein model lead to a greater effect in changes to blood lipids and cytokines (adiponectin and leptin) with an ES of 0.60, 2.14, 0.59, and 0.77, for TC, HDL, adiponectin and leptin respectively.

Also exercise of high intensity (indicted as with RT training intensities ≥75% of 1RM at a training volume of 2–4 sets of 6–10 reps and when free-weight resistance is utilized or ET utilizing interval intensities or a steady-state with intensities ≥70% VO_2max_ or HR_max_) elicited greater effectiveness at inducing changes to body composition, insulin levels, blood lipids, and cytokines (adiponectin, CRP, IL-6), with an ES of 0.49, 0.66, 0.37, 0.50, 0.75, 0.78, 0.75, 0.66, 1.15, and 0.92 for BM, FM, FFM, Insulin, TC, LDL, TG, adiponectin, CRP, and IL-6 respectively. As should be of no surprise, the combination of a high level of training intensity (regardless of method of exercise, ET or RT or ET and RT, or in combination with diet or not) induced a greater effect on the level of energetic imbalance than a lower level of training intensity. When comparisons based on training intensity indicate a clear preference towards use of higher levels of training intensity. Where higher intensity training once again elicited a greater effect in the responses than lower intensities (ES of 0.66, 0.3, 0.42, 1.15, 0.92 for adiponectin, leptin, TNF-α, CRP and IL-6 respectively). And is better than the diet only option for treatment (ES of 0.26, 0.59, 0.29, 0.86, 0.33 for adiponectin, leptin, TNF-α, CRP and IL-6 respectively). Comparison between exercise modalities indicates RT protocols produced a greater ES for changes in adiponectin for higher intensities (ES of 0.74), but not for lower (ES of −1.14) with no differences noted for changes in leptin, relative to ET. Likewise, RT induced a greater ES for changes in IL-6 and CRP relative to ET, at higher (ES of 0.27 and 1.34, respectively) and lower intensities (ES of 0.36 and 0.76, respectively). Furthermore there is an indication for favoring higher intensity RT at an ES of 0.47 for BM, of 0.30 for FM and 0.40 for FFM, respectively to any of the ET protocols, ET and RT combination or lower intensity RT. And favor higher intensity ET at an ES for 0.35 for BM and 0.39 for FM but not for retention of FFM 0.13 relative to lower intensity ET. Where comparisons between exercise intensities within the ET and the RT protocols, indicated favor toward ET (ES of 0.66, 1.13, 0.61, and 0.96 for TC, HDL, LDL, and TG respectively), and RT (ES of 0.85, 0.86, and 0.60 for TC, LDL, and TG respectively).

## Discussion

Given that any change in behavior in highly sedentary individuals who are overfat should result in an immediate effective means for altering both body composition and health status. That occurs regardless of the methods utilized for the adult who is overfat. And given that all studies in publication indicate an ability to produce a positive effect to both body composition and health status. It should not be surprising to find ES across studies that indicate and effective treatment regardless of methodology utilized. Yet, while all treatment options show a favor for effective treatment for inducing changes in body mass. The effectiveness by which the body composition measures changed was highly variable based on the specific methodology being utilized. Moreover, they varied widely in the effectiveness for the biomarkers of health status of the adult who is overfat, Table 2. Moreover, the analysis of ES pooled across studies in aggregate indicate here is that what has been the general classically recommend treatment for overfatness, and associated diseases, may not actually be the most effective. Where the methodological, and sociological, bias towards said programs may be the inherent rationale for continued praise and high recommendation to individuals who are overfat. And may promote the reoccurring cycles of repetitive diets and exercise programs for changes in body morphology and health status [13,14,15,16].

As evident in the fact that classically recommended, and routinely cited in popular press, lower fat diet was less effective for changing any body compositional measures relative to the other dietary only options. With the higher protein diets being more effective than the glycemic controlled diets relative to the lower fat diets. Therefore, should a diet-only intervention be recommended, and in agreement with previous reviews on the topic [61-64], a hypocaloric high-protein/low carbohydrate diet appears to generate the greatest ES for change relative to all hypocaloric, and low fat, diets. This effectiveness appears within diet interventions that utilized a level >1.5 g protein*kg^−1^body mass (>25% of total kcal/d), within the hypocaloric diet with a CI for ES induced always favoring the high protein diet, while not with diets with lower protein, ~1.0 g of protein*kg^−1^BM (<20% of total kcal/d), and higher carbohydrate (regardless of glycemic load) threshold for ES induced a CI_.95_ that crosses into the area of having no effect (i.e. ES ≤ 0) at changing of body composition.

Further, the addition of exercise provided stimulus for responses that are at least as effective as any diet-only method for altering body composition, see Table 2 and Figure 2. And analysis of effectiveness showed preference of favor toward RT rather than the classically recommended ET at being more effective to elicit beneficial changes. When combined with diet, exercise interventions were more effective at inducing responses in body compositional changes than either an exercise, or diet, alone option for intervention. The effectiveness for exercise becomes more pronounced with higher levels of intensity of exercise regardless of the methodology employed (i.e. ET, RT, or combination therein) within the intervention protocol. Additionally, there is a clear delineation between the modes of exercise used and the effectiveness at inducing responses. While heavy recommended by a number of organizations and through a variety of position stands [17,55,65], or stated in previous reviews on the subject [4,7-10,12], as being more effective at inducing changes in body composition the use of a ET alone, or in combination with RT, and in combination with diet interventions were not more effect than the combination of RT with diet, Figure 2.

Within this difference of effectiveness for treatment, diet with RT was not only more effective at altering BM in the most beneficial pattern (i.e. reduction of FM with retention of FFM), without regard to level of training, versus any of the other categorization of the methods for exercise. And when employed at even lower levels of stimulation (e.g., <70% 1RM, single set for at least 12 repetitions, use of pneumatic or selectorized machines, and performance of circuit resistance training) RT provides responses that mimic the ES from ET, or the combination to ET and RT. Where responses mirror each other, whether or not diet is involved in the treatment. And becomes more effective at higher levels of stimulation RT (e.g., >75-80% 1RM for at least 3 sets with repetition ranges of 5-to-10 with 60-to-90 second rest intervals) at inducing changes in body composition that leads to the reduction of BM and FM, while retaining (and in some cases increases of) FFM for the individual who is overfat. Further, ET appeared to have its greatest effect when either in an interval style of ET, or at higher intensities of at ≥75% VO_2max_ (or HR_max/peak_), while not at the traditionally recommended moderate (e.g., 55-75% VO_2max_, or HR_max/peak_) steady-state ET for response to changes in BM and FM, but not for changes in FFM.

There is also the classically held view of the relationship between caloric imbalance and the altering body composition for adults who are overfat. Where if the assumption is correct, there should be a relationship of equivalence in effectiveness for changing caloric balance with body compositional changes between treatment methods. However, based on analysis here, the effectiveness for inducing changes in caloric imbalance does not match the effectiveness to induce body compositional changes for the adult that is overfat. This alternate view to the equation indicates, as previously speculated [19,27], that the issue of overfatness is one that is highly complex. Where there a variety of interconnected factors at play beyond the simplistic caloric balance issue relative to not only body composition but also the alteration of health status for the adult who is overfat. And hints at a possible problem for continually linking these two factors in relationship to changes, not only body compositional changes but also the health status change. As there are number of problem rationales for such an argument, namely changes in hormonal functions related to energetic balance (i.e. leptin, ghrelin) and tightly associated with metabolic markers of exertional stress (i.e. AMPK) [5,12,66,67]. Along with the inherent problem related to measuring the absolute of energetic imbalance that may be incurred from any intervention. Principally that the indicated energetic imbalances are an assumed difference in energetic balance. As very few protocols directly measure the imbalance and no study reviewed on the topic directly measured the energetic shift from either the exercise sessions, the recovery from said sessions. With only a few indicated changes in resting metabolic rate related to either the hypocaloric diet or exercise or combination therein. Thus it becomes troubling that such relationships are continually stated as an absolute as opposed to the assumption that it appears to be. Therefore, it may be more beneficial to discuss intervention methods based on metabolic stress (and demand) rather than on the energetic imbalance, based on the assumed difference, for the adult who is overfat.

While changes to body composition appear to be key in the reinforcement necessary for continual use of the treatment protocols over long periods of time, providing the cheerleader effect for continuation of an intervention. The changes elicited in humoral factors (e.g., hormone/cytokines, blood lipids and biomarkers of inflammation) are necessary for improvement in health status that many have previously discussed in a number of reviews on this topic [18-21,26,31,32,34,42]. As one of the key indicators for metabolic health issues for adults that are overfat is high levels of circulating insulin, it would be expected that an effective therapeutic treatment would elicit reductions in fasting levels of insulin would indicate improvements in metabolic and immune conditions [19,26,28,31-34,42].

In such, there are patterns of responses indicating a spectrum of effectiveness, within and across the various methods of diet, exercise or combination of diet with exercise. As indicated with inducing changes in fasting levels of insulin, where dieting alone is shown to be overall less effective than any of the exercise or diet in combination with exercise modalities. Once again the high-protein (regardless of carbohydrate modification) diet was more effective than the simply having a hypocaloric, or the traditional low fat, diet within the spectrum of diet options examined, ES of 0.49. And is seen even more so when combined with an exercise programs, ES of 0.77. Lending further support to the evolving opinion regarding the employment of higher protein diets for adults who are overfat.

Additionally, exercise was more effective at inducing changes in fasting insulin levels than diet. And in congruence with many of the position stands offered and classically recommend [1,17,55], the use of ET (both alone and in combination with diet or in conjunction of diet and RT) was more effective than RT (either when used alone or in combination with diet) for eliciting changes in insulin. This difference in treatment effectiveness is reversed with incorporation of the high protein/low carbohydrate diet with combination of exercise where RT is more effective than ET, regardless of intensity (ES of 3.5). It should also be noted that the combination of diet with RT was only intervention that provided a pooled therapeutic ES that did not elicit the possibility of no response (i.e. crosses a point of ES = 0) from treatment relative to the control. And not surprisingly, the use of higher-intensity exercise was more effective than lower-intensity exercise without regard to diet selection. While these findings support the use of ET within treatment protocols, there is an indication that RT is a viable option for the adult who is overfat and does not self-select towards an ET mode of exercise [13,15,27,28]. Thus given the findings here, utilizing RT can be a more effective treatment for reversing insulin resistance, as the psychological adherence to the program may provide additional reinforcement for continual use of exercise within a treatment regimen. And when combined with the combination of a high protein/low carbohydrate diet, RT exercise (regardless of level of intensity) can be significantly more effective than the standard ET recommendations.

There is also a spectrum effectiveness to elicit responses in blood lipids form the various treatments indicates trends in the data towards the use of exercise (ET, RT or combination thereof) either alone or in combination with diet for effectiveness of treatment over the use of simple dietary interventions. In which all treatments offer a small degree of effectiveness for altering lipid profiles for the individual who is overfat. With the combination of diet and exercise was more effective than diet alone or exercise alone. And as indicated here, there is a favor toward use of RT is seen with eliciting reductions of blood lipids levels, TC, LDL and TG, relative to either dieting alone, or in combination of diet with ET. Which lends further support toward using RT within the treatment methods. As previously noted, this difference in effectiveness becomes even more pronounced in favor of the higher intensity exercise protocols (regardless of using ET or RT).

This spectrum for a continuum of effectiveness continues as related to the levels of cytokines (e.g., TNF-α, CRP, leptin and adiponectin) related to inflammation and chronic immune response. Where any treatment is able to produce an effective change that leads toward a normal “healthy” range, thus leading to a reduced risk for development of cardiovascular disease and improvements in work capacity and overall health [18,26,32,34,42,68]. However, these responses were highly variable and most of the indications for effectiveness, both as a therapeutic effect and treatment effect, near that point of zero difference in effect (i.e. ES = 0). Most interesting were responses seen in changes to levels of CRP, found in relation to diet alone and diet with RT. Where diet combined with RT induced an almost equal level of effectiveness to that of diet alone. With both indicated as being less effective than the combination of diet with ET or the combination of diet with ET and RT. Indicating a possible metabolic difference between exercise modalities that might induce the differential cardiovascular adaptations noted following these distinct intervention protocols.

Moreover, there are differences in effectiveness noted between RT and ET. This is seen regardless of being utilized alone or with modification to diet, or based on the intensities of training. Based on such stratification there is an indication for the role of the metabolic demand of treatment eliciting differential response to cytokine and adipokine signals that alter whole body metabolism. Where it appears that the better means for prescription of exercise is at the higher levels of training intensities. And when associated with the concept of self-selection toward distinct exercise modes leading to greater utilization [13,15,56], supports the indication for practitioners to recommend and prescribe the use of RT within treatment options that have been speculated about previously [28,47-50]. As incorporating RT may provide the metabolic stimulus to not only the means for improvement of health status but as it may be more readily self-selected lead to longer periods of utilization such activities throughout one’s remaining lifespan as been previously suggested [15,69]. Especially if RT is prescribed at the higher levels of training intensities than what has been previously recommended and closer to what is traditionally utilized for hypertophication responses in lean and active individuals.

While one intention of the study here was to examine the changes in anabolic hormones that have shown reduced levels with overfatness (e.g., testosterone (T) and growth hormone (GH)) in particular relative to the therapeutic interventions of diet, exercise or combination of diet and exercise. There were too few studies that looked at these changes in relation to the treatments that were used, that did not involve a pharmaceutical intervention. From the few studies that examined this change, the relative changes in absolute values note an increase in testosterone and growth hormone that seem to not be related to the intervention used, but instead changes in FM following treatment. While a number of studies have examined the issue in responses acutely either to exercise relative to differences between the normal fat control and the overfat population, or in relation to a pharmaceutical treatment option without use of exercise. Given the current opinions [36,70-72] regarding the role of such hormones in relation to body composition and disease it seems that studying such changes may prove to be a very fruitful avenue for future research in the various intervention programs. Especially given the previously noted changes in GH from hypocaloric diets and within various exercise treatments utilized [73,74]. Which is matched with the changes in levels of T, binding proteins and peripheral receptors for T that are associated with exercise, in particular RT in a fasting state (which should relate well with a hypocaloric model), and may mirror the hormone replacement therapy treatment application for some individuals with this population [45,46,132,75-78]. However, there is limited analysis to speculate either to the extent, beyond expected changes toward normal levels, or time frame for changes within anabolic hormones for adults who are overfat. But given the compatibility of immunological and metabolic profiles between the overfat and the elderly populations, it can be speculated that use of exercise, in particular RT should mimic what has been shown with elderly populations [79-82].

## Conclusions

Analysis of effectiveness of responses both within and between interventions differences for treatment options modalities (e.g., diet, exercise, or combination therein) along with submodality of treatment (e.g., high intensity versus low intensity, high protein/low carbohydrate diets) indicate a continuum of effectiveness. Most importantly is that protocols utilizing exercise were more effective than those that employed just a hypocaloric diet. With the combination of diet with exercise (especially RT) being more effective than diet or diet with ET in reduction of body mass and fat mass while retaining of FFM following treatment. And are at least as effective for changing hormonal levels and blood lipid profiles. Also, while popular ideas suggest the necessity for acute energetic imbalance, there appears to be no relationship between any treatments effectiveness for inducing acute changes in energetic balance with the effectiveness for induced responses to body composition or biomarkers of health from said treatment program. All of which reinforces the idea of a more complex network of factors that influence overall body composition and health issues for the adult who is overfat, and further stresses the idea to focus treatment on generating a metabolic stress to induce chronic endocrinological (and cytokine) changes as opposed to the focus on the kcal/d (kJ/d) ratios of intake to expenditure.

Further, based on ES for responses to RT (in combination with diet, or with diet and ET), one would be able to expect that at the very least 55% of any population of overfat adults should have beneficial responses in all body compositional measures from the incorporation of RT into a treatment play, along with an even greater percentage having a favorable response to altering fasting levels of insulin, total cholesterol, low-density lipoproteins and triglycerides. Additionally, when exercise is utilized at appropriate intensities (i.e. higher levels) both ET and RT provides an effective stimulus to alter TNF-α, CRP, leptin and adiponectin levels that all indicate a reduction in the risk for cardiovascular disease and improved metabolic flexibility for the adult who is overfat. With RT producing a greater level of effectiveness for altering these measures, especially when RT is progressive and periodized with a training volume of 2-to-3 sets at 6-to-10 reps with an intensity of ≥75% 1RM and a rest interval of 60–90 seconds, and utilizes whole body (and free-weight) exercises. And thus indicates that RT should be more readily recommended as an appropriate treatment option to adults who are overfat than what has been recommended currently.

Yet, however the effectiveness of this combination of diet and RT might be for inducing changes, the concept of self-selection of exercise patterns means that some adults who are overfat may select toward protocols of ET for exercise. For those who self-select toward ET, it appears that ET is more effective when performed at high intensity (e.g., ≥70% VO_2max_, or HR_max_) steady-state method or as an interval training style (based on ES calculated gives an expectations of at least 40% of the population showing beneficial responses to intervention). Likewise, some may select away from exercise altogether, which based on overall effectiveness should be discouraged but if utilized as a stand alone intervention, diets can be effective if hypocaloric and comprised of a higher percentage of total caloric intake from protein, with an expectation for at least 55% of the population showing a beneficial response from the intervention.

Lastly, there needs to be further examination of findings noted here. First, related to the ongoing understanding of the anabolic dysregulation that accompanies the situation of being overfat. In this light there is a need to examine the relationship of changes in said hormones based on intervention within populations of individuals who are overfat. Not with simply acute comparison to lean active population, but within the concept of altering levels of anabolic hormones, responses at peripheral tissues and the relative timeframe for seeing such hormonal responses based on the various interventions utilized. And how the impact of periodization and concurrent exercise exposure has on these responses. Second, related to the issues of differential response between genders to identify if there may be a more beneficial response for males versus those for females, and vice versa. Third, based on the current understanding of application of exercise modalities if there are differential responses to programs based on location for intervention and professional associated with overseeing intervention (e.g., in hospital versus out-patient physical therapy clinic versus community health center/gymnasium or for-profit health center/gymnasium). Additionally, and as noted earlier, there needs to be an evaluation of programs and protocols readily available to the populous or utilized within studies for this population. Most exercise programs seem to be highly elaborate for the sake of complexity. In what appears as an effort of marketing the program as being different, as opposed to being elaborate for the sake of progressive periodization. Where the elaboration for periodization of exercise is meant to provide stimulus for continual adaptations within the exerciser. Finally, most programs that have been established based on the idea of energetic imbalance need to be careful with establishing such an idea, as the energetic imbalance is based on an assumption that might not be held in all cases. As changes to not only body composition but also health status comes from manipulation of highly elaborate network of factors that interact, compliment and confound the impact of each other for the adult who is overfat leading to not only body compositional changes, but reversal of the deleterious health outcome of being overfat.

## Abbreviations

D: Diet
ET: Endurance Training
RT: Resistance Training
E + R: Combination of ET and RT
D(E): Diet with ET
D(ER): Diet with combination of ET and RT
D(R): Diet with RT
BM: Body mass (kg)
BMI: Body mass index (kg*m^−3^)
%BF: The percent of body mass comprised on fat mass
FM: Fat mass (kg, or as determined by %body fat)
FFM: Fat-free mass (kg, or as determined by %fat-free mass)
TG: Plasmal triglycerides
TC: Plasmal total cholesterol
HDL: High-density lipoproteins
LDL: Low-density lipoproteins
ES: Effect size as determined by pooled effect size via random effect computation
T2DM: Type 2 diabetes mellitus or metabolic syndrome
T: Testosterone
GH: Growth hormone
CRP: C-reactive protein
TNF-α: Tumor necrotic factor-alpha
VO_2max_: Maximal aerobic capacity
HR_max_: Maximal sustainable heart rate
1RM: Maximal level of resistance for a single repetition (i.e. maximal strength)

## References

[1] Centers for Disease Control and Prevention (CDC). Adult participation in aerobic and muscle-strengthening physical activities--United States, 2011. MMWR Morbidity and mortality weekly report. 2013; 62: 326–330 PMC4604926 23636025

[2] Brown A. Snapshot: U.S. obesity rate ticking up. Gallup. 2013. http://www.gallup.com/poll/163205/snapshot-obesity-rate-ticking.aspx, Accessed, October, 20, 2013.

[3] Mendes E. Americans exercising less in 2013. Gallup. 2013. http://www.gallup.com/poll/163718/americans-exercising-less-2013.aspx, Accessed, October, 20, 2013.

[4] Hansen D, Dendale P, Berger J, van Loon LJC, Meeusen R (2007). The effects of exercise training on fat-mass loss in obese patients during energy intake restriction. Sports Med..

[5] Hainer V, Toplak H, Mitrakou A (2008). Treatment modalities of obesity: what fits whom?. Diabetes Care..

[6] Lee DC, Sui X, Blair SN (2009). Does physical activity ameliorate the health hazards of obesity?. Br J Sports Med..

[7] Strasser B, Spreitzer A, Haber P (2007). Fat loss depends on energy deficit only, independently of the method for weight loss. Ann Nutr Metab..

[8] Varady KA, Bhutani S, Klempel MC, Kroeger CM (2011). Comparison of effects of diet versus exercise weight loss regimens on LDL and HDL particle size in obese adults. Lipids Health Dis..

[9] Votruba SB, Horvitz MA, Schoeller DA (2000). The role of exercise in the treatment of obesity. Nutrition..

[10] Larson-Meyer DE, Redman L, Heilbronn LK, Martin CK, Ravussin E (2010). Caloric restriction with or without exercise: the fitness versus fatness debate. Med Sci Sports Exerc..

[11] Lee S, Kuk JL, Davidson LE, Hudson R, Kilpatrick K, Graham TE (2005). Exercise without weight loss is an effective strategy for obesity reduction in obese individuals with and without Type 2 diabetes. J Appl Physiol..

[12] Williams PT (2001). Health effects resulting from exercise versus those from body fat loss. Med Sci Sports Exerc..

[13] Brock DW, Irving BA, Gower B, Hunter GR (2011). Differences emerge in visceral adipose tissue accumulation after selection for innate cardiovascular fitness. Int J Obesity (2005)..

[14] Eriksson JG (1999). Exercise and the treatment of type 2 diabetes mellitus. An update. Sports Med..

[15] Fogelholm M (2008). How physical activity can work?. Int J Pediatr Obes..

[16] Hooper LE, Foster-Schubert KE, Weigle DS, Sorensen B, Ulrich CM, McTiernan A (2010). Frequent intentional weight loss is associated with higher ghrelin and lower glucose and androgen levels in postmenopausal women. Nutri Res (New York, NY)..

[17] American College of Sports Medicine (ACSM), American Diabetes Association (ADA) (2010). Exercise and type 2 diabetes: American College of Sports Medicine and the American Diabetes Association: joint position statement. Exercise and type 2 diabetes. Med Sci Sports Exerc..

[18] Bruunsgaard H (2005). Physical activity and modulation of systemic low-level inflammation. J Leukoc Biol..

[19] Clark JE (2012). An overview of the contribution of fatness and fitness factors, and the role of exercise, in the formation of health status for individuals who are overweight. J Diab Metab Disord..

[20] Duncan GE (2010). The “fit but fat” concept revisited: population-based estimates using NHANES. Int J Behav Nutr Phys Act..

[21] McAuley PA, Blair SN (2011). Obesity paradoxes. J Sports Sci..

[22] Ioannidis JPA (2005). Why most published research findings are false. PLoS Med..

[23] Pereira TV, Horwitz RI, Ioannidis JPA (2012). Empirical evaluation of very large treatment effects of medical interventions. JAMA..

[24] Bird SP, Tarpenning KM, Marino FE (2005). Designing resistance training programmes to enhance muscular fitness: a review of the acute programme variables. Sports Med..

[25] Kraemer WJ, Ratamess NA (2004). Fundamentals of resistance training: progression and exercise prescription. Med Sci Sports Exerc..

[26] Booth FW, Gordon SE, Carlson CJ, Hamilton MT (2000). Waging war on modern chronic diseases: primary prevention through exercise biology. J Appl Physiol (Bethesda, Md : 1985)..

[27] Clark JE. Role of resistance training in obesity treatments. 1st International Conference on Obesity, OMICS. Philadelphia, PA, 2012.

[28] Clark JE, Goon DT (2015). The roles of resistance training for treatment of obesity related health issue and for changing health status of the individual who is overfat or obese: A review. J Sports Med Phys Fit.

[29] Fantuzzi G (2005). Adipose tissue, adipokines, and inflammation. J Allergy Clin Immunol..

[30] Hood DA, Irrcher I, Ljubicic V, Joseph A-M (2006). Coordination of metabolic plasticity in skeletal muscle. J Exp Biol..

[31] Matheson EM, King DE, Everett CJ (2012). Healthy lifestyle habits and mortality in overweight and obese individuals. J Am Board Fam Med..

[32] Mathur N, Pedersen BK (2008). Exercise as a mean to control low-grade systemic inflammation. Mediators Inflamm..

[33] Müssig K, Remer T, Maser-Gluth C (2010). Brief review: Glucocorticoid excretion in obesity. J Steroid Biochem Mol Biol.

[34] Bastard J-P, Maachi M, Lagathu C, Kim MJ, Caron M, Vidal H (2006). Recent advances in the relationship between obesity, inflammation, and insulin resistance. Eur Cytokine Netw..

[35] Abate N, Haffner SM, Garg A, Peshock RM, Grundy SM (2002). Sex steroid hormones, upper body obesity, and insulin resistance. J Clin Endocrinol Metab..

[36] Brick DJ, Gerweck AV, Meenaghan E, Lawson EA, Misra M, Fazeli P (2010). Determinants of IGF1 and GH across the weight spectrum: from anorexia nervosa to obesity. Eur J Endocrinol..

[37] Dyck DJ (2009). Adipokines as regulators of muscle metabolism and insulin sensitivity. Appl Physiol Nutr Metab..

[38] Gerrits MF, Ghosh S, Kavaslar N, Hill B, Tour A, Seifert EL (2010). Distinct skeletal muscle fiber characteristics and gene expression in diet-sensitive versus diet-resistant obesity. J Lipid Res..

[39] Gualillo O, González-Juanatey JR, Lago F (2007). The emerging role of adipokines as mediators of cardiovascular function: physiologic and clinical perspectives. Trends Cardiovasc Med..

[40] Guay AT (2009). The emerging link between hypogonadism and metabolic syndrome. J Androl..

[41] Mammi C, Calanchini M, Antelmi A, Cinti F, Rosano GMC, Lenzi A (2012). Androgens and adipose tissue in males: a complex and reciprocal interplay. Int J Endocrinol..

[42] Maya-Monteiro CM, Bozza PT (2008). Leptin and mTOR: partners in metabolism and inflammation. Cell Cycle..

[43] Mohr BA, Bhasin S, Link CL, O’Donnell AB, McKinlay JB (2006). The effect of changes in adiposity on testosterone levels in older men: longitudinal results from the Massachusetts Male Aging Study. Eur J Endocrinol..

[44] Nannipieri M, Bonotti A, Anselmino M, Cecchetti F, Madec S, Mancini E (2007). Pattern of expression of adiponectin receptors in human adipose tissue depots and its relation to the metabolic state. Int J Obes (Lond)..

[45] Saad F, Aversa A, Isidori AM, Gooren LJ (2012). Testosterone as potential effective therapy in treatment of obesity in men with testosterone deficiency: a review. Current Diab Reviews..

[46] Saad F, Gooren LJ. The role of testosterone in the etiology and treatment of obesity, the metabolic syndrome, and diabetes mellitus type 2. J Obes. 2011; doi:10.1155/2011/471584. 10.1155/2011/471584 PMC2931403 20847893

[47] Balducci S, Zanuso S, Nicolucci A, Fernando F, Cavallo S, Cardelli P (2009). Anti-inflammatory effect of exercise training in subjects with type 2 diabetes and the metabolic syndrome is dependent on exercise modalities and independent of weight loss. Nutr Metab Cardiovasc Dis.

[48] Pedersen BK, Febbraio MA (2012). Muscles, exercise and obesity: skeletal muscle as a secretory organ. Nat Rev Endocrinol..

[49] Shaibi GQ, Roberts CK, Goran MI (2008). Exercise and insulin resistance in youth. Exerc Sport Sci Rev..

[50] Tresierras MA, Balady GJ (2009). Resistance training in the treatment of diabetes and obesity: mechanisms and outcomes. J Cardiopulm Rehabil Prev..

[51] Bouchard DR, Soucy L, Sénéchal M, Dionne IJ, Brochu M (2009). Impact of resistance training with or without caloric restriction on physical capacity in obese older women. Menopause..

[52] Ahima RS (2011). Digging deeper into obesity. J Clin Invest..

[53] Bacon L, Aphramor L (2011). Weight science: evaluating the evidence for a paradigm shift. Nutr J..

[54] Duncan DT, Wolin KY, Scharoun-Lee M, Ding EL, Warner ET, Bennett GG (2011). Does perception equal reality? Weight misperception in relation to weight-related attitudes and behaviors among overweight and obese US adults. Int J Behav Nutri Phys Act..

[55] Lichtenstein AH, Appel LJ, Brands M, Carnethon M, Daniels S, American Heart Association Nutritional Committee (2006). Diet and lifestyle recommendations revision 2006: a scientific statement from the American Heart Association Nutrition Committee. Circulation..

[56] Garland T, Schutz H, Chappell MA, Keeney BK, Meek TH, Copes LE (2011). The biological control of voluntary exercise, spontaneous physical activity and daily energy expenditure in relation to obesity: human and rodent perspectives. J Exp Biol..

[57] Booth FW, Laye MJ (2009). Lack of adequate appreciation of physical exercise’s complexities can pre-empt appropriate design and interpretation in scientific discovery. J Physiol Lond..

[58] Becker B (1988). Synthesizing stanardized mean-changing measures. Br J Math Stat Psychol..

[59] Krieger JW (2010). Single vs. multiple sets of resistance exercise for muscle hypertrophy: a meta-analysis. J Strength Cond Res.

[60] Morris S, DeShon RP (2002). Combining effect size estimates in meta-analysis with repeated measures and independent-groups designs. Psychol Methods..

[61] Mettler S, Mitchell N, Tipton KD (2010). Increased protein intake reduces lean body mass loss during weight loss in athletes. Med Sci Sports Exerc..

[62] Volek JS, Westman EC. Very-low-carbohydrate weight-loss diets revisited. Cleve Clin J Med. 2002;69:849–53. 856–848 passim. 10.3949/ccjm.69.11.849 12430970

[63] Volek JS, Sharman MJ, Forsythe CE (2005). Modification of lipoproteins by very low-carbohydrate diets. J Nutr..

[64] Phinney SD (2004). Ketogenic diets and physical performance. Nutri Metabol..

[65] Centers for Disease Control and Prevention (CDC) (2011). School health guidelines to promote healthy eating and physical activity. MMWR Recomm Rep..

[66] Friedman JM, Halaas JL (1998). Leptin and the regulation of body weight in mammals. Nature..

[67] Meier U, Gressner AM (2004). Endocrine regulation of energy metabolism: review of pathobiochemical and clinical chemical aspects of leptin, ghrelin, adiponectin, and resistin. Clin Chem..

[68] Buford TW, Cooke MB, Willoughby DS (2009). Resistance exercise-induced changes of inflammatory gene expression within human skeletal muscle. Eur J Appl Physiol..

[69] Fortier MS, Duda JL, Guerin E, Teixeira PJ (2012). Promoting physical activity: development and testing of self-determination theory-based interventions. Int J Behav Nutri Phys Act..

[70] Li C, Ford ES, Li B, Giles WH, Liu S (2010). Association of testosterone and sex hormone-binding globulin with metabolic syndrome and insulin resistance in men. Diabetes Care..

[71] Tishova Y, Kalinchenko SY (2009). Breaking the vicious circle of obesity: the metabolic syndrome and low testosterone by administration of testosterone to a young man with morbid obesity. Arq Bras Endocrinol Metabol..

[72] Vikan T, Schirmer H, Njølstad I, Svartberg J (2010). Low testosterone and sex hormone-binding globulin levels and high estradiol levels are independent predictors of type 2 diabetes in men. Eur J Endocrinol..

[73] Berryman DE, Christiansen JS, Johannsson G, Thorner MO, Kopchick JJ (2008). Role of the GH/IGF-1 axis in lifespan and healthspan: lessons from animal models. Growth Horm IGF Res..

[74] Liu H, Bravata DM, Olkin I, Nayak S, Roberts B, Garber AM, Hoffman AR (2007). Systematic review: the safety and efficacy of growth hormone in the healthy elderly. Ann Intern Med..

[75] Bhattacharya RK, Khera M, Blick G, Kushner H, Nguyen D, Miner MM (2011). Effect of 12 months of testosterone replacement therapy on metabolic syndrome components in hypogonadal men: data from the Testim Registry in the US (TRiUS). BMC Endocr Disord..

[76] Kelly DM, Jones TH (2013). Testosterone: a metabolic hormone in health and disease. J Endocrinol..

[77] Mekala KC, Tritos NA (2009). Effects of recombinant human growth hormone therapy in obesity in adults: a meta analysis. J Clin Endocrinol Metab..

[78] Scacchi M, Pincelli AI, Cavagini F (1999). Growth hormone in obesity. Int J Obes..

[79] Consitt LA, Bloomer RJ, Wideman L (2007). The effect of exercise type on immunofunctional and traditional growth hormone. Eur J Appl Physiol..

[80] Craig BW, Brown R, Everhart J (1989). Effects of progressive resistance training on growth hormone and testosterone levels in young and elderly subjects. Mech Ageing Dev..

[81] Hagerman FC, Walsh SJ, Staron RS, Hikida RS, Gilders RM, Murray TF (2000). Effects of high-intensity resistance training on untrained older men. I. Strength, cardiovascular, and metabolic responses. J Gerontol A Biol Sci Med Sci.

[82] Häkkinen K, Pakarinen A, Kraemer WJ, Newton RU, Alen M (2000). Basal concentrations and acute responses of serum hormones and strength development during heavy resistance training in middle-aged and elderly men and women. J Gerontol A Biol Med Sci..

[83] Ahmadizad S, Haghighi AH, Hamedinia MR (2007). Effects of resistance versus endurance training on serum adiponectin and insulin resistance index. Eur J Endocrinol..

[84] Anderssen SA, Carroll S, Urdal P, Holme I (2007). Combined diet and exercise intervention reverses the metabolic syndrome in middle-aged males: results from the Oslo Diet and Exercise Study. Scand J Med Sci Sports..

[85] Ara I, Perez-Gomez J, Vicente-Rodriguez G, Chavarren J, Dorado C, Calbet JAL (2006). Serum free testosterone, leptin and soluble leptin receptor changes in a 6-week strength-training programme. BrJ Nutri..

[86] Ballor DL, Harvey-Berino JR, Ades PA, Cryan J, Calles-Escandon J (1996). Contrasting effects of resistance and aerobic training on body composition and metabolism after diet-induced weight loss. Metab Clin Exp..

[87] Ballor DL, Katch VL, Becque MD, Marks CR (1988). Resistance weight training during caloric restriction enhances lean body weight maintenance. Am J Clin Nutri..

[88] Borg P, Kukkonen-Harjula K, Fogelholm M, Pasanen M (2002). Effects of walking or resistance training on weight loss maintenance in obese, middle-aged men: a randomized trial. Int J Obes Relat Metab Disord..

[89] Brehm BJ, Seeley RJ, Daniels SR, D’Alessio DA (2003). A randomized trial comparing a very low carbohydrate diet and a calorie-restricted low fat diet on body weight and cardiovascular risk factors in healthy women. J Clin Endocrinol Metab..

[90] Brochu M, Malita MF, Messier V, Doucet E, Strychar I, Lavois J-M (2009). Resistance training does not contribute to improving the metabolic profile after a 6-month weight loss program in overweight and obese postmenopausal women. J Clin Endocrinol Metab..

[91] Bryner RW, Ullrich IH, Sauers J, Donley D, Hornsby G, Kolar M (1999). Effects of resistance vs. aerobic training combined with an 800 calorie liquid diet on lean body mass and resting metabolic rate. J Am Coll Nutr.

[92] Campbell WW, Haub MD, Wolfe RR, Ferrando AA, Sullivan DH, Apolzan JW (2009). Resistance training preserves fat-free mass without impacting changes in protein metabolism after weight loss in older women. Obesity (Silver Spring)..

[93] Christiansen T, Paulsen SK, Bruun JM, Pedersen SB, Richelsen B (2010). Exercise training versus diet-induced weight-loss on metabolic risk factors and inflammatory markers in obese subjects: a 12-week randomized intervention study. Am J Physiol Endocrinol Metab..

[94] Cuff DJ, Meneilly GS, Martin A, Ignaszewski A, Tildesley HD, Frohlich JJ (2003). Effective exercise modality to reduce insulin resistance in women with type 2 diabetes. Diabetes Care..

[95] Donnelly JE, Pronk NP, Jacobsen DJ, Pronk SJ, Jakicic JM (1991). Effects of a very-low-calorie diet and physical-training regimens on body composition and resting metabolic rate in obese females. AmJ Clin Nutri..

[96] Donnelly JE, Sharp T, Houmard J, Carlson MG, Hill JO, Whately JE (1993). Muscle hypertrophy with large-scale weight loss and resistance training. Am J Clin Nutri..

[97] Donnelly JE, Hill JO, Jacobsen DJ, Potteiger J, Sullivan DK, Johnson SL (2003). Effects of a 16-month randomized controlled exercise trial on body weight and composition in young, overweight men and women: the Midwest Exercise Trial. Arch Intern Med..

[98] Dunstan DW, Daly RM, Owen N, Jolley D, DeCourten M, Shaw J, Zimmet P (2002). High-intensity resistance training improves glycemic control in older patients with type 2 diabetes. Diabetes Care..

[99] Fisher G, Hyatt TC, Hunter GR, Oster RA, Desmond RA, Gower BA (2011). Effect of diet with and without exercise training on markers of inflammation and fat distribution in overweight women. Obesity (Silver Spring, Md)..

[100] Foster GD, Wyatt HR, Hill JO, McGuckin BG, Brill C, Mohammed BS (2003). A randomized trial of a low-carbohydrate diet for obesity. N Engl J Med..

[101] Geliebter A, Maher MM, Gerace L, Gutin B, Heymsfield SB, Hashim SA (1997). Effects of strength or aerobic training on body composition, resting metabolic rate, and peak oxygen consumption in obese dieting subjects. Am J Clin Nutri..

[102] Goodpaster BH, Delany JP, Otto AD, Kuller L, Vockley J, South-Paul JE (2010). Effects of diet and physical activity interventions on weight loss and cardiometabolic risk factors in severely obese adults: a randomized trial. JAMA..

[103] Hallsworth K, Fattakhova G, Hollingsworth KG, Thoma C, Moore S, Taylor R (2011). Resistance exercise reduces liver fat and its mediators in non-alcoholic fatty liver disease independent of weight loss. Gut..

[104] Hammer RL, Barrier CA, Roundy ES, Bradford JM, Fisher AG (1989). Calorie-restricted low-fat diet and exercise in obese women. Am J Clin Nutri..

[105] Hill JO, Sparling PB, Shields TW, Heller PA (1987). Effects of exercise and food restriction on body composition and metabolic rate in obese women. Am J Clin Nutri..

[106] Hill JO, Schlundt DG, Sbrocco T, Sharp T, Pope-Cordle J, Stetson B (1989). Evaluation of an alternating-calorie diet with and without exercise in the treatment of obesity. Am J Clin Nutri..

[107] Ho SS, Dhaliwal SS, Hills AP, Pal S (2012). The effect of 12 weeks of aerobic, resistance or combination exercise training on cardiovascular risk factors in the overweight and obese in a randomized trial. BMC Public Health..

[108] Ibáñez J, Izquierdo M, Martínez-Labari C, Ortega F, Grijalba A, Forga L (2010). Resistance training improves cardiovascular risk factors in obese women despite a significative decrease in serum adiponectin levels. Obesity (Silver Spring)..

[109] Irving BA, Weltman JY, Patrie JT, Davis CK, Brock DW, Swift D (2009). Effects of exercise training intensity on nocturnal growth hormone secretion in obese adults with the metabolic syndrome. J Clin Endocrinol Metabol..

[110] Josse AR, Atkinson SA, Tarnopolsky MA, Phillips SM (2011). Increased consumption of dairy foods and protein during diet- and exercise-induced weight loss promotes fat mass loss and lean mass gain in overweight and obese premenopausal women. J Nutr..

[111] Kempen KP, Saris WH, Westerterp KR (1995). Energy balance during an 8-wk energy-restricted diet with and without exercise in obese women. Am J Clin Nutri..

[112] Kerksick C, Thomas A, Campbell B, Taylor L, Wilborn C, Marcello B (2009). Effects of a popular exercise and weight loss program on weight loss, body composition, energy expenditure and health in obese women. Nutrition Metabol..

[113] Klimcakova E, Polak J, Moro C, Hejnova J, Majercik M, Viguerie N (2006). Dynamic strength training improves insulin sensitivity without altering plasma levels and gene expression of adipokines in subcutaneous adipose tissue in obese men. J Clin Endocrinol Metab..

[114] Kraemer WJ, Volek JS, Clark KL, Gordon SE, Puhl SM, Koziris LP (1999). Influence of exercise training on physiological and performance changes with weight loss in men. Med Sci Sports Exerc..

[115] Larsen TM, Dalskov S-M, van Baak M, Jebb SA, Papadaki A, Pfeiffer AFH (2010). Diets with high or low protein content and glycemic index for weight-loss maintenance. N Engl J Med..

[116] Layman DK, Boileau RA, Erickson DJ, Painter JE, Shiue H, Sather C, Christou DD (2003). A reduced ratio of dietary carbohydrate to protein improves body composition and blood lipid profiles during weight loss in adult women. J Nutri..

[117] Layman DK, Evans E, Baum JI, Seyler J, Erickson DJ, Boileau RA (2005). Dietary protein and exercise have additive effects on body composition during weight loss in adult women. J Nutri..

[118] Maiorana A, O’Driscoll G, Goodman C, Taylor R, Green D (2002). Combined aerobic and resistance exercise improves glycemic control and fitness in type 2 diabetes. Diabetes Res Clin Pract..

[119] Marks BL, Ward A, Morris DH, Castellani J, Rippe JM (1995). Fat-free mass is maintained in women following a moderate diet and exercise program. Med Sci Sports Exerc..

[120] Moreira MM, Souza HPC, Schwingel PA, Sá CKC, Zoppi CC (2008). Effects of aerobic and anaerobic exercise on cardiac risk variables in overweight adults. Arq Bras Cardiol..

[121] Nicklas BJ, Ambrosius W, Messier SP, Miller GD, Penninx BWJH, Loeser RF (2004). Diet-induced weight loss, exercise, and chronic inflammation in older, obese adults: a randomized controlled clinical trial. Am J Clin Nutri..

[122] Oberbach A, Tönjes A, Klöting N, Fasshauer M, Kratzsch J, Busse MW (2006). Effect of a 4 week physical training program on plasma concentrations of inflammatory markers in patients with abnormal glucose tolerance. Eur J Endocrinol..

[123] Olson TP, Dengel DR, Leon AS, Schmitz KH (2007). Changes in inflammatory biomarkers following one-year of moderate resistance training in overweight women. Int J Obesity (2005)..

[124] Pavlou KN, Steffee WP, Lerman RH, Burrows BA (1985). Effects of dieting and exercise on lean body mass, oxygen uptake, and strength. Med Sci Sports Exerc..

[125] Phinney SD, O’Connell M, Danforth E (1988). Effects of aerobic exercise on energy expenditure and nitrogen balance during very low calorie dieting. Metabolism..

[126] Polak J, Klimcakova E, Moro C, Viguerie N, Berlan M, Hejnova J (2006). Effect of aerobic training on plasma levels and subcutaneous abdominal adipose tissue gene expression of adiponectin, leptin, interleukin 6, and tumor necrosis factor alpha in obese women. Metab Clin Exp..

[127] Pritchard JE, Nowson CA, Wark JD (1997). A worksite program for overweight middle-aged men achieves lesser weight loss with exercise than with dietary change. J Am Diet Assoc..

[128] Racette SB, Schoeller DA, Kushner RF, Neil KM (1995). Exercise enhances dietary compliance during moderate energy restriction in obese women. Am J Clin Nutri..

[129] Rice B, Janssen I, Hudson R, Ross R (1999). Effects of aerobic or resistance exercise and/or diet on glucose tolerance and plasma insulin levels in obese men. Diabetes Care..

[130] Rolland C, Hession M, Broom I (2011). Effect of weight loss on adipokine levels in obese patients. Diabetes Metab Syndrome Obes..

[131] Ross R, Janssen I, Dawson J, Kungl A-M, Kuk JL, Wong SL (2004). Exercise-induced reduction in obesity and insulin resistance in women: a randomized controlled trial. Obes Res..

[132] Ryan AS, Nicklas BJ, Berman DM, Elahi D (2003). Adiponectin levels do not change with moderate dietary induced weight loss and exercise in obese postmenopausal women. Int J Obes Relat Metab Disord..

[133] Schjerve IE, Tyldum GA, Tjønna AE, Stølen T, Loennechen JP, Hansen HEM (2008). Both aerobic endurance and strength training programmes improve cardiovascular health in obese adults. Clin Sci..

[134] Sheu WH-H, Chang T-M, Lee W-J, Ou H-C, Tseng L-N, Lang H-F (2008). Effect of weight loss on proinflammatory state of mononuclear cells in obese women. Obesity (Silver Spring, Md)..

[135] Sigal RJ, Kenny GP, Boulé NG, Wells GA, Prud’homme D, Fortier M (2007). Effects of aerobic training, resistance training, or both on glycemic control in type 2 diabetes: a randomized trial. Ann Intern Med..

[136] Slentz CA, Bateman LA, Willis LH, Shields AT, Tanner CJ, Piner LW (2011). ffects of aerobic vs. resistance training on visceral and liver fat stores, liver enzymes, and insulin resistance by HOMA in overweight adults from STRRIDE AT/RT. Am J Physiol Endocrinol Metab.

[137] Tjønna AE, Lee SJ, Rognmo Ø, Bye A, Haram PM, Loennechen JP (2008). Aerobic interval training versus continuous moderate exercise as a treatment for the metabolic syndrome: a pilot study. Circulation..

[138] Tokmakidis SP, Zois CE, Volaklis KA, Kotsa K, Touvra A-M (2004). The effects of a combined strength and aerobic exercise program on glucose control and insulin action in women with type 2 diabetes. Eur J Appl Physiol..

[139] Trapp EG, Chisholm DJ, Freund J, Boutcher SH (2008). The effects of high-intensity intermittent exercise training on fat loss and fasting insulin levels of young women. Int J Obes (Lond)..

[140] Volpe SL, Kobusingye H, Bailur S, Stanek E (2008). Effect of diet and exercise on body composition, energy intake and leptin levels in overweight women and men. J Am Coll Nutr..

[141] Wang P, Holst C, Andersen MR, Astrup A, Bouwman FG, van Otterdijk S (2011). Blood profile of proteins and steroid hormones predicts weight change after weight loss with interactions of dietary protein level and glycemic index. PLoS One..

[142] Watkins LL, Sherwood A, Feinglos M, Hinderliter A, Babyak M, Gullette E (2003). Effects of exercise and weight loss on cardiac risk factors associated with syndrome X. Arch Intern Med..

[143] Wycherley TP, Noakes M, Clifton PM, Cleanthous X, Keogh JB, Brinkworth GD (2010). A high-protein diet with resistance exercise training improves weight loss and body composition in overweight and obese patients with type 2 diabetes. Diabetes Care..

