# Erratum to: Diet, exercise or diet with exercise: comparing the effectiveness of treatment options for weight-loss and changes in fitness for adults (18–65 years old) who are overfat, or obese; systematic review and meta-analysis

**DOI:** 10.1186/s40200-015-0204-8

**Published:** 2015-09-28

**Authors:** James E. Clark

**Affiliations:** Division of Mathematics, Science, and Health Careers; Department of Science, Manchester Community College, Manchester, 06045-1046 CT USA

Unfortunately, the original version of this article [1] contained an error. The presentation of Fig. 2 (Fig. 1 here) contained an incorrect labelling of the x-axis. The corrected figure can be found included below.

**Fig. 1:**
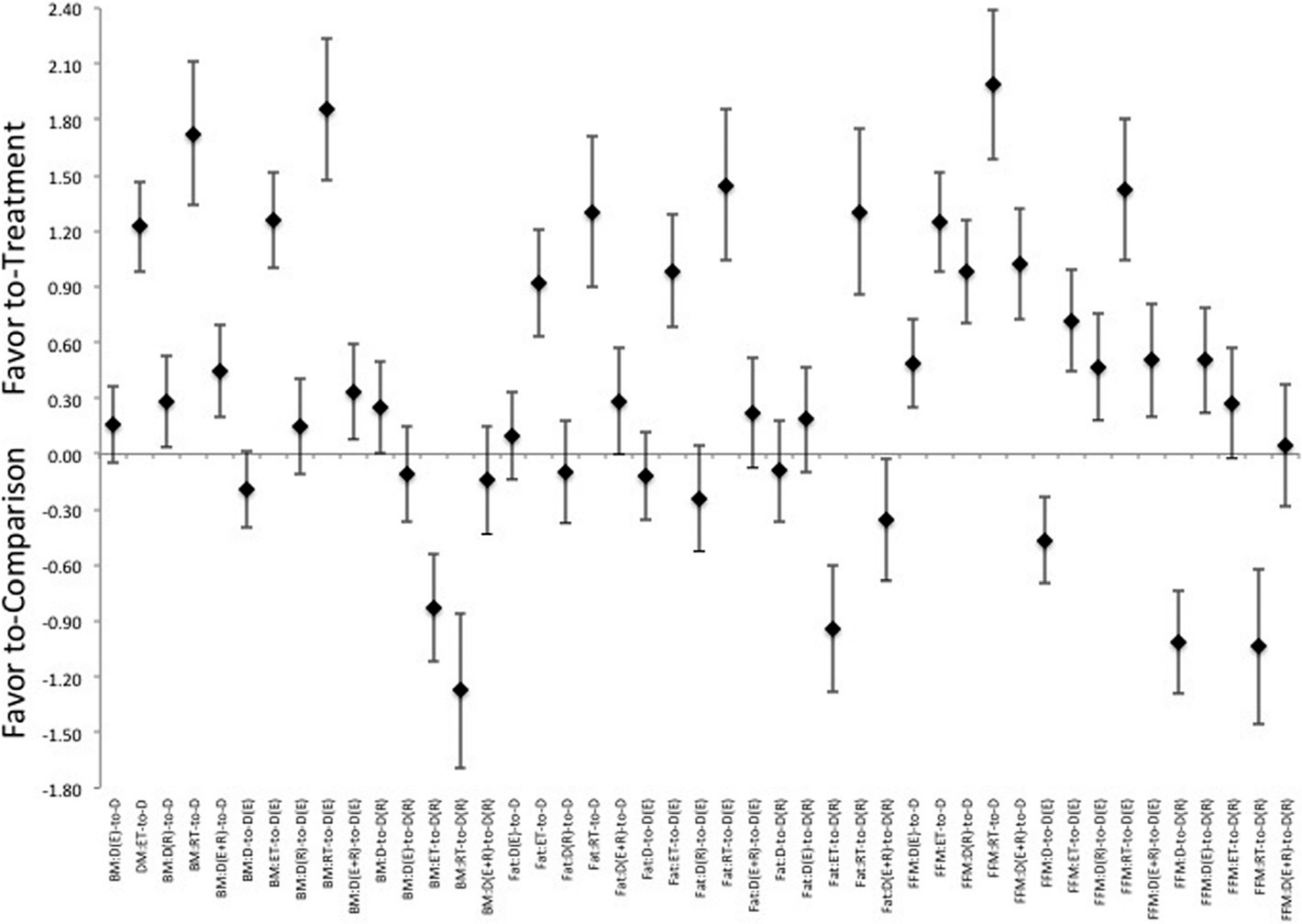
Description of the pooled ES for treatment response and the range of CI for ES between intervention (versus diet alone or versus diet with combination of ET, or versus diet with combination of RT) methods for changes in either Body Mass (BM), Fat Mass (Fat), and Fat-Free Mass (FFM). Note that the comparisons are labeled as “treatment-to-comparison”, with D indicating diet-only, D(E) indicating diet with ET, D(R) indicating diet with RT, D(E + R) indicating diet with ET and RT, ET indicating ET-only, and RT indicating RT-only for the various intervention methods within the comparisons
